# Molecular phylogenetic study of *Scleria* subgenus *Hypoporum* (Sclerieae, Cyperoideae, Cyperaceae) reveals several species new to science

**DOI:** 10.1371/journal.pone.0203478

**Published:** 2018-09-27

**Authors:** Kenneth Bauters, Paul Goetghebeur, Pieter Asselman, Kenny Meganck, Isabel Larridon

**Affiliations:** 1 Botanic Garden Meise, Meise, Belgium; 2 Ghent University, Department of Biology, Research Group Spermatophytes, Campus Ledeganck, Ghent, Belgium; 3 Royal Museum for Central Africa, Tervuren, Belgium; 4 Royal Botanic Gardens, Kew, Richmond, Surrey, United Kingdom; Sichuan University, CHINA

## Abstract

*Scleria* subgen. *Hypoporum* (Cyperaceae), with 68 species, is the second largest subgenus in *Scleria*. Species of this pantropically distributed subgenus generally occur in seasonally or permanently wet grasslands or on shallow soils over sandstone or lateritic outcrops, less often they can be found in (open) woodlands. Previous studies established the monophyly of the subgenus, but the relationships between the species remained uncertain. In this study, DNA sequence data of 61 taxa of *Scleria* subgen. *Hypoporum*, where possible represented by multiple accessions from across their distributional range, were obtained for four molecular markers: the coding chloroplast marker *ndhF*, the chloroplast intron *rps16* and the nuclear ribosomal regions ETS and ITS. Phylogenetic trees were constructed using Bayesian inference and maximum likelihood approaches. A species tree was constructed to summarise the results. The results indicate the existence of three sections: the monotypic, pantropically occurring, *Scleria* sect. *Lithospermae*, a new section from central and south America containing two species, and *Scleria* sect. *Hypoporum*, also pantropically distributed, containing the remainder of the species of the subgenus. Relationships in the latter section are not fully resolved. However, three or four different clades can be distinguished supported by some morphological characters. Our results indicate at least six new species in *Scleria* sect. *Hypoporum*. The new section and species are described in a taxonomical treatment. Their morphology is compared with (morphologically) closely related species.

## Introduction

**Fig 1:**
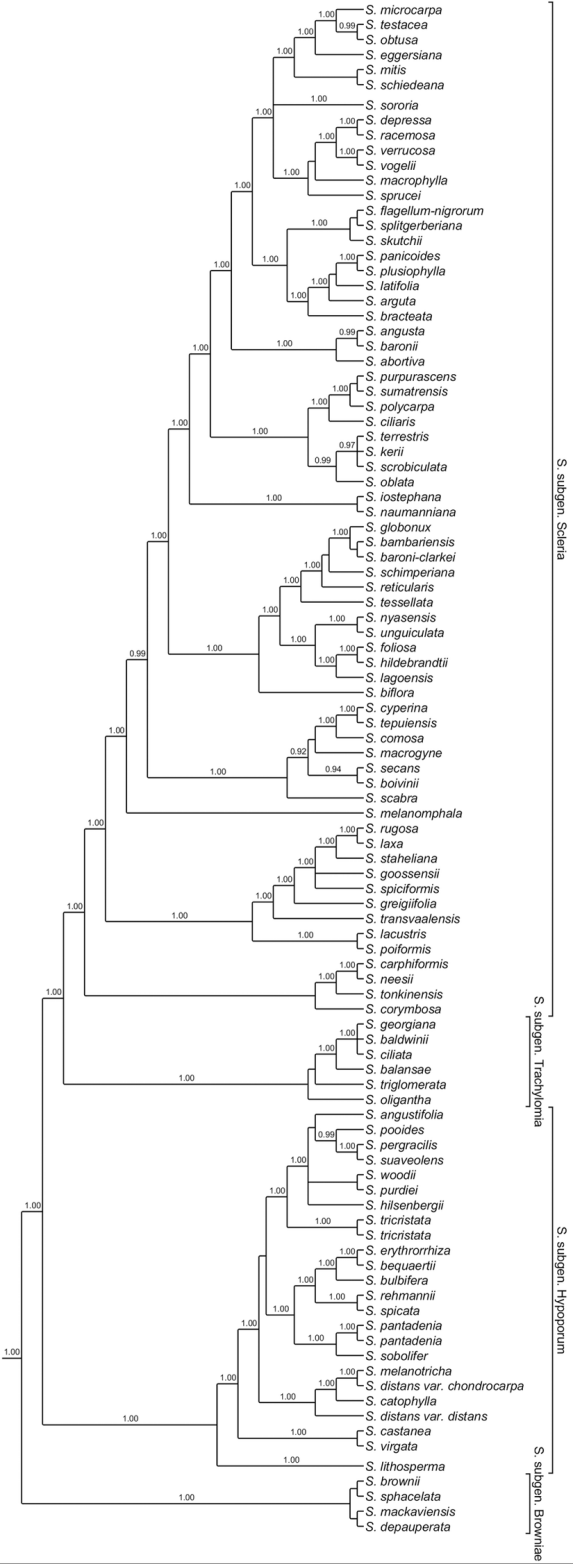
Phylogenetic summary of the genus *Scleria*. Based on Bayesian analysis of Bauters et al. [1]. Posterior probabilities are displayed above the branches. Subgenera are displayed on the tree.

The genus *Scleria* P.J.Bergius is with its ca. 250 species one of the major genera of the sedge family (Cyperaceae) [1, 2]. The genus is placed in the monotypic tribe Sclerieae and has a primarily pantropical distribution, locally extending into warm-temperate regions [1, 3]. Species of *Scleria* occur in open places in forest, grasslands, road- and riversides, swamps, etc. The genus was recently shown to comprise four subgenera and 15 sections [1]. *Scleria* subgen. *Hypoporum* is with its 68 species the second largest subgenus (Fig 1). Its centre of diversity is tropical Africa, with at least 32 species in Zambia alone [4]. The subgenus also frequently occurs in Central and South America [5], with a few species native to North America and one species extending to the southernmost of Canada [5]. From Asia and Oceania, only two species are known, *S*. *lithosperma* (L.) Sw. and *S*. *pergracilis* (Nees) Kunth [6]. The large number of species known from Zambia (over 60 species of *Scleria*) might be attributed to the late Edward Armitage Robinson, who spent 13 years in Zambia collecting with a main interest in *Scleria* [7]. He described 16 new species from Zambia [4, 8] of which nine are now placed in *S*. subgen. *Hypoporum*. His work gives a strong indication that other tropical African regions are probably undercollected, and therefore, we can only guess how many species of *Scleria* are still to be discovered.

Species of *Scleria* subgen. *Hypoporum* are commonly found in seasonally or permanently wet grasslands, on shallow soils over sandstone or lateritic outcrops and less often in (open) woodlands [3, 4, 5, 9]. The hypogynium is one of the main distinguishing characters for other subgenera of *Scleria* [1] and its absence or reduction is the main identifier for species of *S*. subgen. *Hypoporum*. The reduced hypogynium is a synapomorphic feature of the subgenus. Species of *Scleria* subgen. *Hypoporum* are either annuals, or have perennial subterranean organs and annual aerial parts [10]. Inflorescences are terminal glomerate-spicate or paniculate with exception of a few species that also have axillary glomerate-spicate or paniculate side branches. They exist mostly of linear spikes of distant, sessile spikelet clusters, either of the unbranched Hypoporum s.str. or the branched (paniculate) Hypoporum s.l. type [1]. This spike of spikelet clusters is also called a glomerate-spicate inflorescence. Spikelets are androgynous with basally one or two empty distichous glumes and one lateral pistillate flower followed by several spirally arranged staminate flowers. Nutlets vary in size and wall ornamentation but lack or have a reduced hypogynium (possible exceptions in e.g. *S*. *bequaertii*, *S*. *laxiflora* and *S*. *procumbens*).

*Hypoporum* Nees was first described at genus level based on the absence of a hypogynium (“perigynium nullum”) [11]. In his treatment, Nees listed 12 species, however no species descriptions were provided [11]. Some authors (e.g. [12, 13, 14, 15]) agreed with Nees and accepted *Hypoporum* at genus level, while others (e.g. [16, 17, 18, 19]) treated it as a synonym of *Scleria*. Clarke was the first who used the taxon at subgenus level as *S*. subgen. *Hypoporum* [20]. Since then, there has been little discussion about the infrageneric nature of *Hypoporum*.

Despite the rather simple overall morphology with few morphological distinguishing characters, more than 200 names have been published [2, 21] for the 68 currently accepted species of *Scleria* subgen. *Hypoporum* [1, 22]. The inconstant appearance of the nutlet ornamentation also led to a lot of confusion [9]. Nutlets occasionally range from smooth to tuberculate and sometimes to lacunose or having other surface markings in the same collection and even on the same individual plant [5, 9]. This caused some authors to create new species based on uniquely the ornamentation of the nutlet (e.g. *S*. *spondylogona* Nelmes [9]). Another point of disagreement is the absence or reduction of the hypogynium. Some authors (e.g. [10]) stated that the absence of a hypogynium reflects the ancestral state in *Scleria*, thus referring to it as a primitive character state, while others (e.g. [2]) hypothesised that the hypogynium is not absent but reduced and is therefore a derived character state. Recently, it was shown that the vestigial hypogynium is indeed a derived state [1].

Clarke further divided *Scleria* subgen. *Hypoporum* into four sections [23]: *S*. sect. *Pergraciles* C.B.Clarke, *S*. sect. *Hirtellae* (C.B.Clarke) C.B.Clarke, *S*. sect. *Lithospermae* (C.B.Clarke) C.B.Clarke and *S*. sect. *Corymbosae* Pax. This was one of the few attempts to further classify *Hypoporum* species. Their relatively few distinguishing characters make it difficult to group them in morphological sections. Also, in the previous study of Bauters et al. [1], the relationships in *S*. subgen. *Hypoporum* were not resolved, and therefore, no attempts were made to further divide the subgenus into sections [1]. However, it was shown that species belonging to *S*. sect. *Corymbosae* are members of *S*. subgen. *Scleria* and that both *S*. *baldwinii* and *S*. *georgiana* belong to *S*. subgen. *Trachylomia* and not to *S*. subgen. *Hypoporum* as suggested by Core [5]. Excluding these, the subgenus was supported as monophyletic with two early diverging lineages [1]. The first diverging branch contains only one species, *S*. *lithosperma*. This is one of four *S*. subgen. *Hypoporum* species that have an inflorescence consisting of terminal and lateral panicles, the others being *S*. *bequaertii* De Wild., *S*. *laxiflora* Gross and *S*. *procumbens* E.A.Robinson. Inflorescences of *S*. *lithosperma*, however, are not contracted into a glomerate-spicate form. Next, a clade with *S*. *castanea* Core and *S*. *virgata* (Nees) Steud. was retrieved [1]. In the species of this clade, spikelets often form loose spikelet clusters. The remainder of *S*. subgen. *Hypoporum* were found to form a well-supported clade but relations between species were not clear [1].

Based on nuclear (ETS, ITS) and chloroplast (*ndhF* gene, *rps16* intron) sequence data, and with an enlarged sample size compared to the dataset of Bauters et al. [1], this study aims to obtain a well-resolved molecular phylogenetic hypothesis of *Scleria* subgen. *Hypoporum*. Based on the molecular results, the classification of the subgenus is updated, and a new section and several new species are formally described.

## Material and methods

### Taxon sampling

Sampling for this study consists of 174 samples, representing 61 taxa of *Scleria* subgen. *Hypoporum* and four outgroup taxa. S1 Table lists all taxa included with voucher information, geographical details and GenBank accession numbers. Samples for DNA extractions were collected from herbarium specimens in BR, GENT, L, MO, NY, US (abbreviations according to Thiers, continuously updated [24]). Additionally, silica gel-dried samples from the U.S.A. and Zambia and living material from the Ghent University Botanical Garden were used. Where possible, leaf material was sampled. As outgroup, five specimens of *S*. subgen. *Browniae* were included in the sampling. *Scleria* subgen. *Browniae* is sister to all other *Scleria* [1]. *Scleria* subgen. *Hypoporum* on its turn, is sister to the remainder of *Scleria*. Species of subgen. *Browniae* were choosen as outgroup based on their basal position within *Scleria*.

### DNA extraction, amplification, and sequencing

After tissue homogenisation (Mixer Mill, Retsch, Haan, Germany), total DNA was extracted from 5–20 mg of dried material using innuPREP Plant DNA Kit (Analytik Jena, Jena, Germany) following the manufacturer’s protocol. Amplifications were performed in volumes of 25 μl containing a GeneAmp 10× PCR buffer with 100 mM Tris-HCL, pH 8.3, 500 mM MgCl_2_ 0.01% (w/v) gelatin (Applied Biosystems, Thermo Fisher Scientific, Waltham, Massachusetts, U.S.A.), dNTP solution of 10 mM (5-prime), ampliTaq DNA polymerase (Lifetechnologies, Carlsbad, California, U.S.A.) with 5 U/μl, primer solution with a concentration of 10 μM, 1μl bovine serum albumin (BSA) and 1 μl of unquantified DNA. For this study, 17 primers were used of which the *rps16* primers were newly adapted from previously used primers (see Table 1 for all primers used). The following primer combinations were used initially: ETS1f & 18SR, ITSA & ITS4, ndhFA & ndhF-D1, rps16F & rps16R (Table 1). Where necessary, internal primers were used (Table 1). For ITS, one additional primer was used (ITSsef17) when first attempts failed to result in usable PCR products. Initial denaturation was set to 3 min at 95°C. After this polymerase chain reaction (PCR) was performed for 30 (ETS and ITS) and 35 (*ndhF* and *rps16*) cycles of denaturation (0:30 min at 95 (ETS, ITS), 0:45 min at 95° (*ndhF*, *rps16*), primer annealing (0:30 min at 52° (ETS, ITS), 0:45 min at 50° (*ndhF*, *rps16*)), and primer extension (1:30 min at 72° (ETS, ITS), 1:00 min at 72°C (*ndhF*, *rps16*)). Finally an elongation period of 7 min at 72°C ended the reaction. The PCR products were electrophoresed on 1% agarose gels in 1× Tris-acetate-EDTA (TAE) buffer (pH 8.0) and stained with ethidium bromide to confirm a single product. Afterwards, the cleaned PCR products were sent to Macrogen Europe (Amsterdam, Netherlands) for sequencing on ABI3730XL machines using the same primers as in the PCR reactions.

**Table 1:** Primers used in this study. Primer combinations used: ITSA(ITSsef17)–ITS4, internal: ITSA(ITSsef17)–ITS-C, ITS-D–ITS4; ETS1f–18SR.

Primer name	Primer sequence (5’–3’)	Reference
ITS		
ITS-A	GGAAGGAGAAGTCGTAACAAGG	Blattner & al. [25]
ITSsef17	ACGAATTCATGGTCCGGTGAAGTGT	Sun & al. [26]
ITS-D	CTCTCGGCAACGGATATCTCG	Blattner & al. [25]
ITS-C	GCAATTCACACCACGTATCGY	Blattner & al. [25]
ITS4	TCCTCCGCTTATTGATATGC	White & al. [27]
ETS		
ETS1f	CTGTGGCGTCGCATGAGT TG	Starr et al. [28]
18SR	AGCAAGCATATGACTACTGGCAGG	Starr et al. [28]
*ndhF*		
*ndhF-A*	TATGGTTACCTGATGCCATGGA	Hinchliff & al. [29]
*ndhFIR1*	AAAAGCTGTTARTCCYGC	Bauters & al. [1]
*ndhFIF2*	GCBTGTTTCTGGTCTAAAGATG	Bauters & al. [1]
*ndhFD1*	CTATRTAACCRCGATTATATGACCAA	Hinchlif & al. [29]
*rps16*		
*rps16F_ox*	GTGGTAGAAAGCAACGTGCGACTT	Oxelman & al. [30]
*rps16R_ox*	TCGGGATCGAACATCAATTGCAAC	Oxelman & al. [30]
*rps16F_in1*	GTRGAACGGGAGTGAATTYTT	Bauters & al. [1]
*rps16R_in2*	CTTCGGGGACCTTTAATCCTT	Bauters & al. [1]

### Sequence alignment and phylogenetic reconstruction

Sequences were read into Sequencher v.5.0.1 (Gene Codes Corporation, Ann Arbor, Michigan, U.S.A.) and sequence ends were trimmed automatically. After this, contigs were constructed and loaded into PhyDE v.0.9971 [31]. Sequences were aligned using MAFFT v.7.215 [32, 33] with “maxiterate” and “tree rebuilding number” set to 100 (long run), afterwards, alignments were checked manually. For *ndhF*, sequences were also converted into amino acids using PhyDE to verify that no stop-codons occurred in one of the sequences. Insertions and deletions were coded following the simple indel coding scheme of Simmons & Ochoterena [34] available in SeqState v.1.4.1 [35].

Phylogenetic analyses were carried out on six different sets of data: (1) ETS data, (2) ITS data, (3) concatenated nuclear data, (4) *ndhF* data, (5) *rps16* data, and (6) concatenated chloroplast data. Phylogenetic trees for ITS and ETS are largely congruent, as are those for the two chloroplast markers *ndhF* and *rps16*, although visual inspection revealed several conflicts between the results of the nuclear and chloroplast datasets. For this reason, the nuclear and chloroplast datasets were not concatenated, but data from the two genomes were analysed separately. Tree topologies were searched for using maximum likelihood (ML) and Bayesian inference (BI) methods. Appropriate models were chosen using jModelTest v.2.1.6 [36], with the Bayesian information criterion (BIC) selected. jModelTest selected GTR+G for ETS and *rps16*, GTR+G+I for ITS as best fitting model. For *ndhF*, the alignment was loaded into PartitionFinder v.1.1.1 [37]. The best partition scheme grouped the first and second codon positions of *ndhF* into one partition with GTR+G as best model; the third codon position was in a second partition with GTR+G+I as best fitting model.

ML analyses for all datasets were performed in RAxML v.7.2.8 [38]. Partitions were inserted were needed and a separate partition was created for the binary indel matrix. The model was set accordingly and the bootstrap analysis was set to 1000 replicates. Indels were analysed as binary data using a gamma model.

BI gene tree analyses were conducted using MrBayes v.3.2 [39]. Four runs were conducted for 30 million generations, sampling every 1000^th^ generation. The results were reviewed in Tracer v.1.5 [40] to check for convergence and to obtain burn-in values. The first 25% of trees were discarded as burn-in, with remaining trees used to construct a 50% majority-rule consensus tree. Trees were checked manually for incongruences. jModelTest and MrBayes were run on CIPRES (https://www.phylo.org/index.php/; [41]). Resulting trees were visualised and modified in TreeGraph2 v.2.0.52 [42]. For trees resulting both from the concatenated nuclear and concatenated chloroplast datasets, a summarizing tree was created in TreeGraph2. Here, multiple accessions for the same species were removed to make interpretation easier.

To estimate a species tree, we used a coalescent-based approach on all four markers. The estimation of a species tree was performed in *BEAST [43] implemented in the BEAST v1.6.2 program package [36]. To perform the analyses in *BEAST, species with only one sequence, or species lacking sequences for both nuclear or both chloroplast markers were excluded (S1 Table). A BEAST xml file was generated in BEAUti v1.6.2. In *BEAST, one MCM chain was run for 100.000.000 generations, sampling every 10.000 generations. The burn-in value was set at 10.000, and a majority rule consensus tree was generated from the remaining trees. Substitution models were the same as in the single marker Bayesian analyses. The species tree was visualized in TreeGraph2.

### Morphology, habitat and distribution

Morphological observations and details on habitat and distribution given in the descriptions are based on herbarium specimens and associated data. Besides examining the holo-, iso- and paratypes of the newly discovered species, many specimens of morphologically closely allied taxa were studied in detail at GENT, BR, K, L, MO, NY, US and UZL (abbreviations according to Thiers, continuously updated).

Species comparative measurements were taken with a Kyowa, Model SZM stereomicroscope. Additionally, to investigate micromorphological nutlet characters that are potentially systematically informative for the genus, low-vacuum Scanning Electron Microscopy (Hitachi Tabletop Miscroscope TM-1000) pictures of the nutlets were taken at the VIB Department of Plant Systems Biology, Ghent University.

### Nomenclature

The new taxon names generated as part of this study satisfy the requirements of the International Code of Nomenclature for algae, fungi, and plants, and are hereby effectively published. In addition, they have been submitted to The International Plant Names Index (IPNI), from where they will be made available to the Global Names Index (http://gni.globalnames.org/). The IPNI LSIDs will resolve and the associated information viewed through any standard web browser by appending the LSID contained in this publication to the prefix http://ipni.org/. The online version of this work is archived and available from the following digital repositories: PubMed Central, LOCKSS, and Ghent University Academic Bibliography.

## Results

### Phylogenetic reconstruction

Sequence alignments contained 158 ETS, 154 ITS, 148 *ndhF* and 140 *rps16* sequences representing 61 different species of which 57 are in *Scleria* subgen. *Hypoporum*. The concatenated nuclear (ETS + ITS) alignment contained 170 sequences and the concatenated chloroplast (*ndhF* + *rps16*) alignment 159 sequences. In all analyses, *S*. subg. *Hypoporum* is supported as monophyletic.

### Nuclear dataset

**Fig 2:**
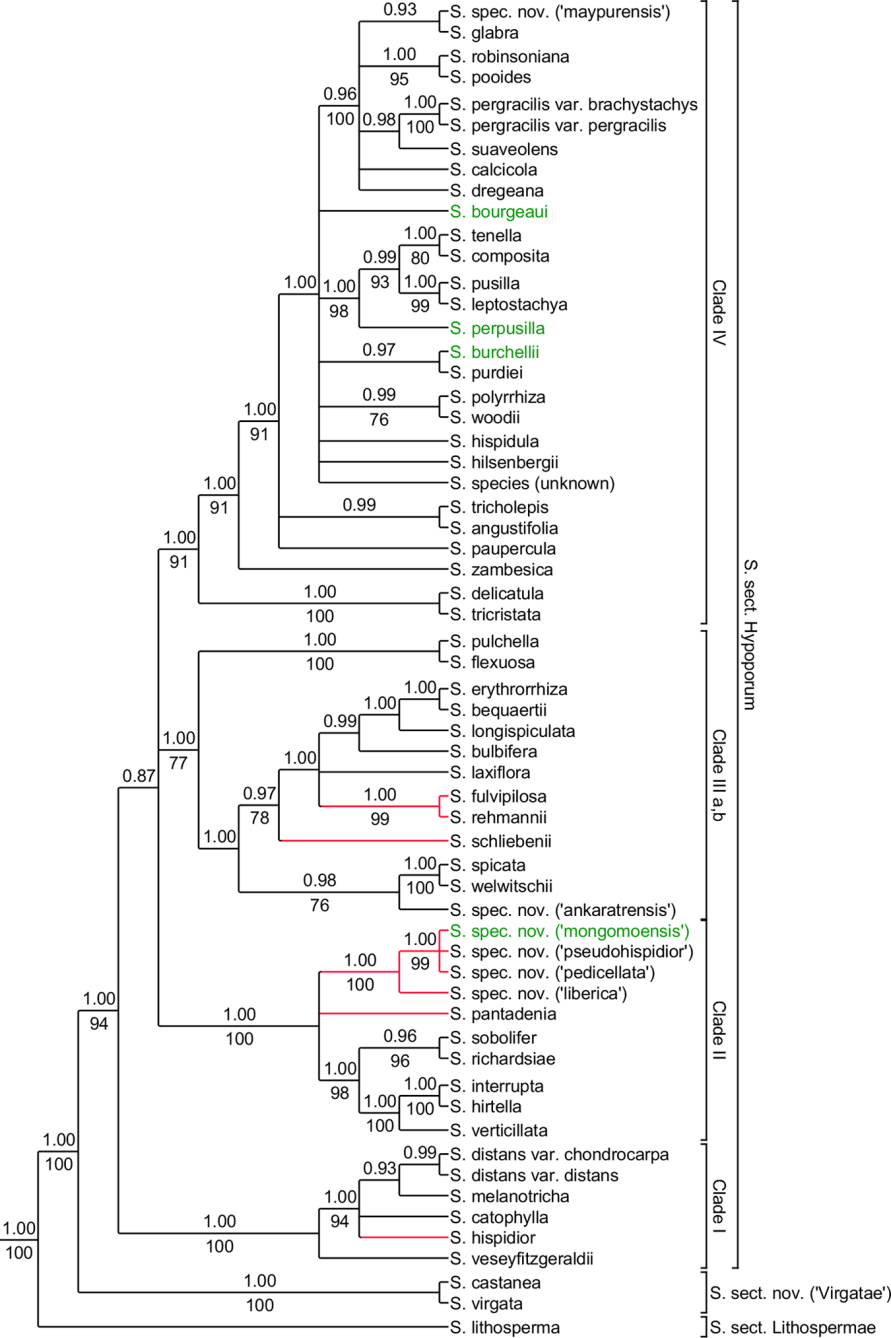
Summary tree of concatenated ETS and ITS analysis. 50% majority rule consensus tree based on concatenated nuclear dataset. Posterior probabilities obtained from BI indicated on the respective branches when equal or higher than 0.90, bootstrap values equal or higher than 75% displayed under the branches. Branches in red indicate differences with the results of the chloroplast dataset. Taxa displayed in green represent samples absent from the chloroplast analyses.

Since the ITS (S1 and S2 Figs) and ETS (S3 and S4 Figs) analyses were largely congruent, their concatenated analysis is also similar. No differences are found between BI (S5 Fig) and ML (S6 Fig) analyses. The results are summarised in Fig 2. *Scleria lithosperma* is sister to all other species of *S*. subgen. *Hypoporum*, and the clade including *S*. *castanea* and *S*. *virgata* is sister to the core *S*. subgen. *Hypoporum* clade. In the core *S*. subgen. *Hypoporum* clade, four different clades can be recognised but relationships between the clades are not clear. Clade I is well-supported and contains the African species of *Scleria* characterised by the conspicuous reddish-black hairs on the glumes and bracts: *S*. *catophylla* C.B.Clarke, *S*. *distans* Poir., *S*. *hispidior* (C.B.Clarke) Nelmes, *S*. *melanotricha* Hochst. ex A.Rich. and *S*. *veseyfitzgeraldii* E.A.Robinson. Clade II (1.00 PP, 100 BS) contains the Tanzanian species *S*. *pantadenia* Meganck & Bauters, a clade with 3 new species from Western Africa (*S*. *spec*. *nov*. *(‘pedicellata’)*, *S*. *spec*. *nov*. *(‘liberica’)*, *S*. *spec*. *nov*. *(‘mongomoensis’)*) and one from East Africa (*S*. *spec*. *nov*. *(‘pseudohispidior’)*) and a clade with three American species (*S*. *hirtella* Sw., *S*. *interrupta* Rich. and *S*. *verticillata* Muhl. ex Willd.) and two African relatives (*S*. *richardsiae* E.A.Robinson and *S*. *sobolifer* E.F.Franklin). Clade III can be divided into two subclades based on the results of the ETS analyses were Clade IIIb is separated from IIIa. In the concatenated nuclear dataset both Clade IIIa and IIIb are united into Clade III (a,b). This is an almost all African clade with one American species, *S*. *spicata* (Spreng.) J.F.Macbr., a close relative of the African *S*. *welwitschii* C.B.Clarke. Relationships between species in Clade IIIa,b are well resolved. *Scleria flexuosa* Boeckeler and *S*. *pulchella* Ridl. form a sister clade to all other members of Clade IIIa,b (1.00 PP, 77 BS). We will use the name Clade III when referring to Clade IIIa,b. Next, a clade with *S*. *spicata*, *S*. *welwitschii* and a species new to science from Madagascar is found (0.98 PP, 76 BS). The largest group of Clade IIIa,b contains *S*. *schliebenii* Gross as sister to the other species (0.97 PP, 78 BS): *S*. *bequaertii*, *S*. *bulbifera* Hochst. ex A.Rich., *S*. *erythrorrhiza* Ridl., *S*. *fulvipilosa* E.A.Robinson, *S*. *laxiflora*, *S*. *longispiculata* Nelmes and *S*. *rehmannii* C.B.Clarke. Relationships between these species vary depending on the analysis. The largest clade of core *Scleria* subg. *Hypoporum* is Clade IV. Within this clade, *Scleria tricristata* Meganck & Bauters and *S*. *delicatula* Nelmes are sister to all other species (1.00 PP, 91 BS). Further relationships in this clade remain unclear. However, most American species, group together in a supported clade (1.00 PP, 98 BS): *S*. *composita* (Nees) Boeckeler, *S*. *leptostachya* Kunth, *S*. *pusilla* Pilg. and *S*. *tenella* Kunth. Only *S*. *perpusilla* Cherm. is not found in the Americas but in Madagascar. A new species is also revealed in Clade IV.

### Chloroplast dataset

**Fig 3:**
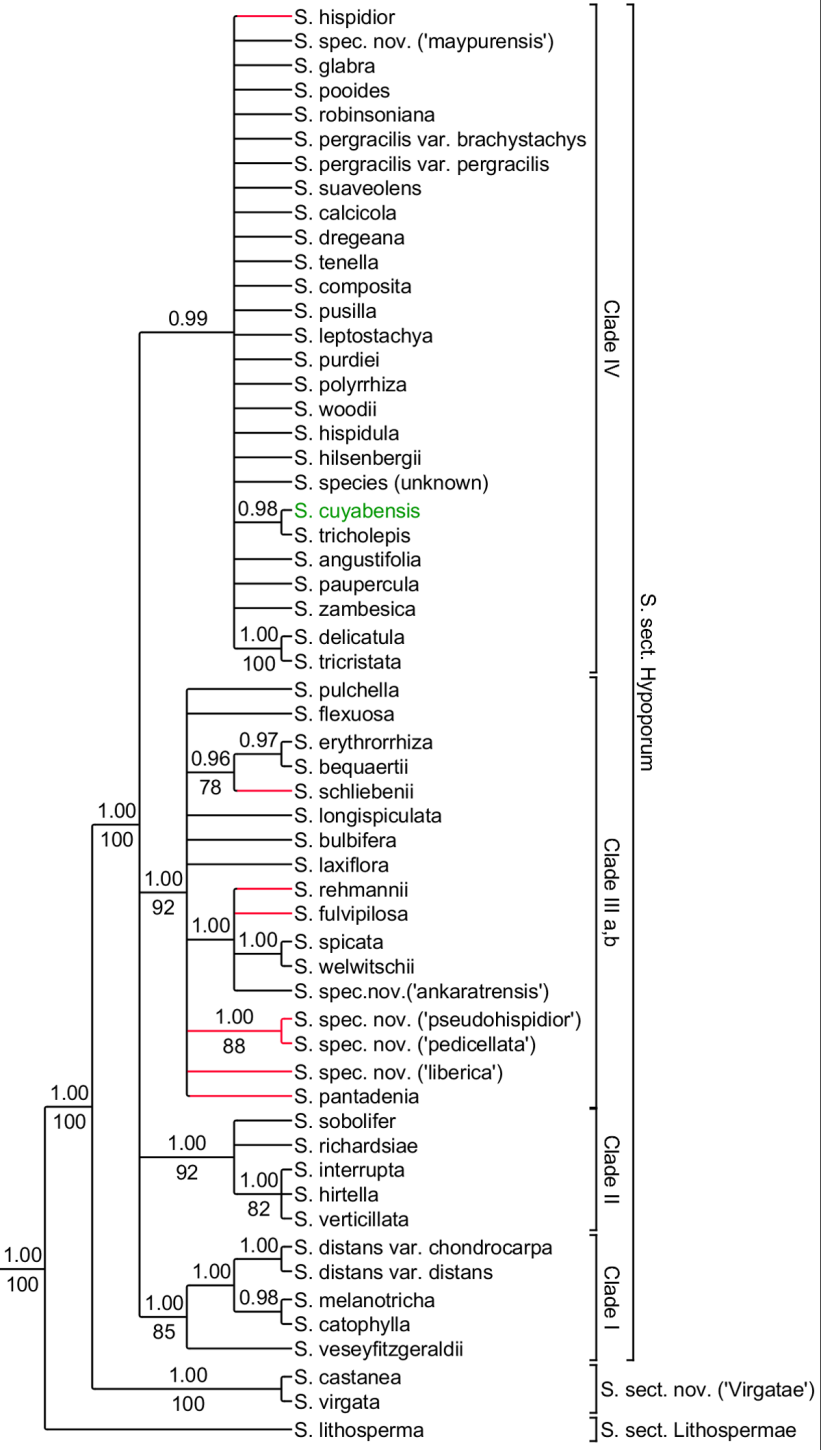
Summary tree of concatenated *ndhF* and *rps16* analysis. 50% majority rule consensus tree based on concatenated chloroplast dataset. Posterior probabilities obtained from BI indicated on the respective branches when equal or higher than 0.90, bootstrap values equal or higher than 75% displayed under the branches. Branches in red indicate differences with the results of the chloroplast dataset. Taxa displayed in green represent samples absent from the chloroplast analyses.

Both the *ndhF* (S7 and S8 Figs) and *rps16* (S9 and S10 Figs) dataset were largely congruent. The majority rule consensus tree of the BI (S11 Fig) and ML (S12 Fig) analyses of the combined chloroplast dataset are congruent. Fig 3 displays the summarised results of the *ndhF* and *rps16* analysis. *Scleria lithosperma* is sister to all other *S*. subgen. *Hypoporum* species (1.00 PP, 100 BS). Next, the clade including *S*. *castanea* and *S*. *virgata* is sister to the core *S*. subgen. *Hypoporum* clade (*S*. sect. *Hypoporum*) (1.00 PP, 100 BS). Within *S*. sect. *Hypoporum*, four different clades are recognised. Clade I corresponds with Clade I of the nuclear dataset (see above). Clade II (1.00 PP, 92 BS) contains only *S*. *hirtella*, *S*. *interrupta*, *S*. *richardsiae*, *S*. *sobolifer* and *S*. *verticillata*. The remaining species, found in Clade II in the nuclear analyses (*S*. *spec*. *nov*. *(‘liberica’)*, *S*. *spec*. *nov*. *(‘pedicellata’)*, *S*. *spec*. *nov*. *(‘pseudohispidior’)* and *S*. *pantadenia*), are included in Clade III. Within Clade III a polytomy is found and relationships between species are unclear. Clade IV is recovered in both analyses but the relationships between species are not resolved within this clade. Differences with the nuclear dataset are limited. The main difference is the generally lower support values obtained based on the chloroplast data. *Scleria hispidior* is included in Clade I in the nuclear analyses and in Clade IV in the chloroplast analyses. *S*. *spec*. *nov*. *(‘liberica’)*, *(‘pedicellata’) and (‘pseudohispidior’)* are included in Clade II for the nuclear dataset and in Clade III in the chloroplast dataset.

### Species tree analysis

**Fig 4:**
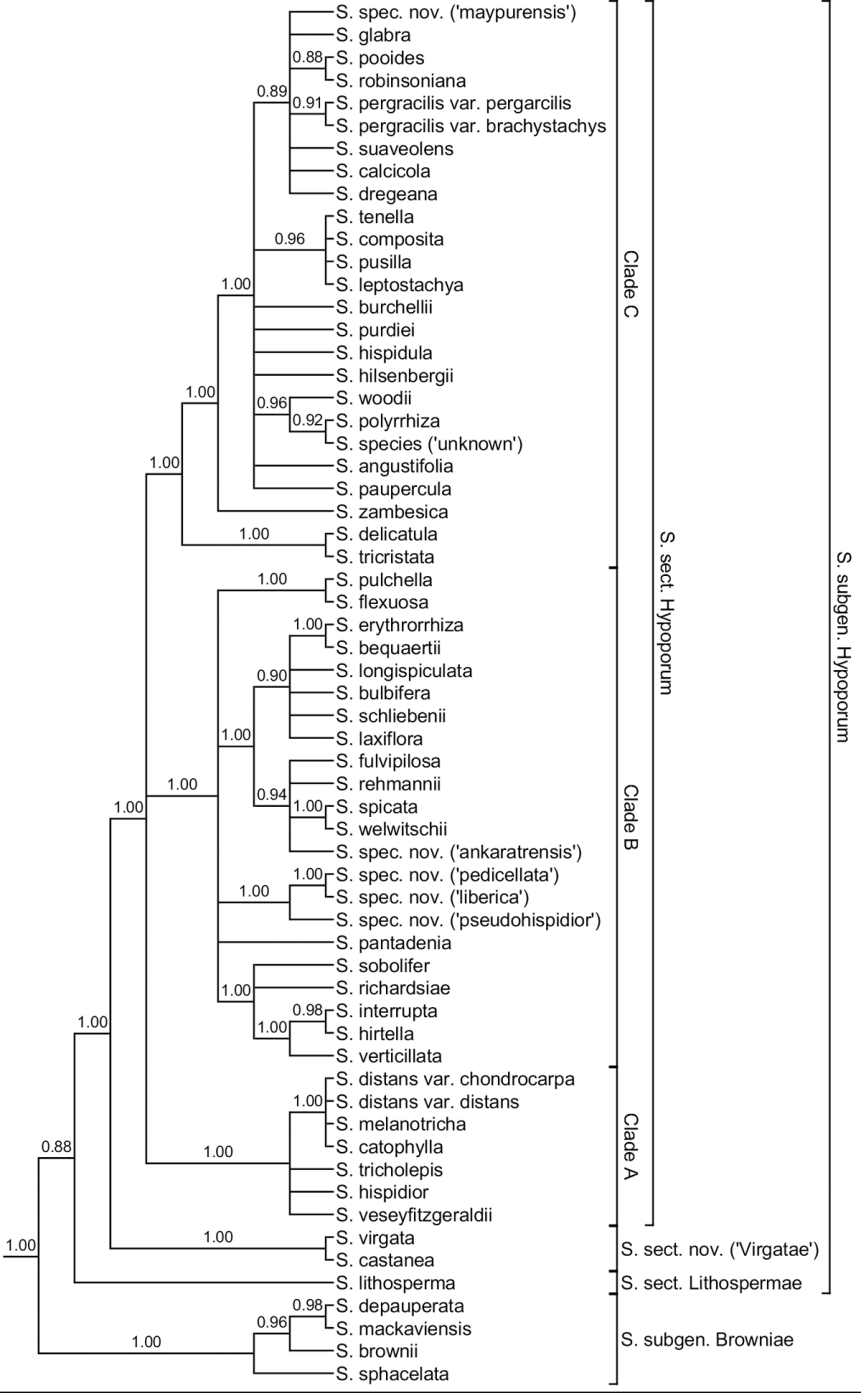
Estimated species tree with coalescent approaches using two nuclear markers, ETS and ITS and two chloroplast markers, *ndhF* and *rps16*. Only posterior probabilities above 0.90 are displayed above the branches.

A species tree was constructed using *BEAST (Fig 4). This analysis supports the monophyly of *Scleria* subgen. *Hypoporum* (1.00 PP). As in all previous analyses, the monotypic *S*. sect. *Lithospermae* is sister to all other species of *S*. subgen. *Hypoporum* (0.88 PP). The all American clade including *S*. *castanea* and *S*. *virgata* is sister to *S*. sect. *Hypoporum*. Within *S*. sect. *Hypoporum*, three major clades are recovered. However, relationships between these clades are not resolved. Clade A is similar to Clade I from BI and ML analyses above. It contains only African species characterised by reddish-black hairs on the glumes and bracts. Only *S*. *distans* var. *distans* also occurs in the Americas. The second clade, Clade B (1.00 PP), combines Clade II and Clade IIIa,b from previous analyses. Clade C (1.00) is similar to Clade IV of previous analyses. Within this clade, *S*. *tricristata* and *S*. *delicatula* form the sister clade to all other species. Next, *S*. *zambesica* E.A.Robinson is sister to the remaining species of this clade. Relationships between other species are unclear.

The results of the *BEAST analysis differ slightly from the results from the nuclear and chloroplast datasets. *Scleria* sect. *Hypoporum* contains only three major clades instead of 4: Clade II and III of the single and concatenated gene analyses are merged into one clade (Clade B). Resolution of relationships between clades and/or species is generally lower in the *BEAST analysis.

### Species new to science

Several species included in this study are new to science. *Scleria spec*. *nov*. *(‘ankaratrensis’)* was recovered in a polytomy with *S*. *fulvipilosa* and *S*. *rehmannii* in the ITS analyses (S1 and S2 Figs) within Clade III. A different position of this species within Clade III was recovered in both ETS analyses. *Scleria spec*. *nov*. *(‘ankaratrensis’)* was found as sister species to *S*. *spicata* and *S*. *welwitschii* (S3 and S4 Figs, 0.99 PP, 80 BS). When concatenating ITS and ETS the close relation with *S*. *spicata* and *S*. *welwitschii* was confirmed (Figs 2, S5 and S6: 0.98 PP, 76 BS). In both chloroplast marker analyses, *S*. *spec*. *nov*. *(‘ankaratrensis’)* was recovered in a clade with *S*. *fulvipilosa*, *S*. *rehmannii*, *S*. *spicata* and *S*. *welwitschii*. In the results of the *ndhF* analyses as sister to these four species (S7 and S8 Figs: 1.00 PP, 98 BS) while in the results of the *rps16*, the concatenated chloroplast and the *Beast analyses it is found in a polytomy with above mentioned species (Figs 3 and 4; S9, S10, S11 and S12 Figs)). Geographically and morphologically this Malagasy species can be clearly separated from the above mentioned species. Morphologically it closely resembles *S*. *hirtella* and *S*. *tricholepis* with which it is compared under ‘Taxonomic Treatment’.

*Scleria spec*. *nov*. *(‘maypurensis’)* was in the ITS and *rps16* analyses recovered in the polytomy of Clade IV. No DNA sequence was obtained for ETS and *ndhF*. No close relationship with another species was revealed. Based on the distinct morphology of the nutlet (see ‘Taxonomic Treatment’) this species is considered as new to science. Morphologicaly this species looks similar to *S*. *verticillata* with which it is compared under ‘Taxonomic Treatment’. In the *Beast analysis, *S*. *spec*. *nov*. *(‘maypurensis’)* was placed in a polytomy within Clade C (Fig 4).

*Scleria spec*. *nov*. *(‘liberica’)*, *S*. *spec*. *nov*. *(‘mongomoensis’)*, *S*. *spec*. *nov*. *(‘pedicellata’) and S*. *spec*. *nov*. *(‘pseudohispidior’)* were found as a separate clade within Clade II for the nuclear analyses. *Scleria spec*. *nov*. *(‘liberica’)* was recoverd as sister to the other three species in the ITS, ETS and concatenated nuclear analyses (S1 and S2 Figs: 1.00 PP, 79 BS; S3 and S4 Figs: 1.00 PP and 100 BS; Figs 2, S5 and S6: 1.00 PP, 100 BS). In the same analyses *S*. *spec*. *nov*. *(‘mongomoensis’)*, *S*. *spec*. *nov*. *(‘pedicellata’) and S*. *spec*. *nov*. *(‘pseudohispidior’)* were always placed within a polytomy, however, morphologically all three species are easily separated. For the chloroplast analyses no sequences were obtained for *S*. *spec*. *nov*. *(‘mongomoensis’)*. In both the *ndhF* and *rps16* analyses, *S*. *spec*. *nov*. *(‘pedicellata’)* is found as sister species of *S*. *spec*. *nov*. *(‘pseudohispidior’)* (S7 and S8 Figs: 1.00 PP, 96 BS and S9 and S10 Figs: 1.00 PP, 88 BS). *Scleria spec*. *nov*. *(‘liberica’)* is placed within a polytomy in Clade III in the chloroplast analyses (Figs 3, S7, S8, S11 and S12). For the *Beast analysis, *S*. *spec*. *nov*. *(‘mongomoensis’)* was not included. The other three new species were found within clade B, forming a separate monophyletic clade (Fig 4: 1.00 PP). *Scleria spec*. *nov*. *(‘pseudohispidior’)* was found as sister species of both *S*. *spec*. *nov*. *(‘pedicellata’)* and *S*. *spec*. *nov*. *(‘liberica’)* (Fig 4: 1.00 PP). Morphologically, *S*. *spec*. *nov*. *(‘liberica’)* resembles *S*. *interrupta* and *S*. *tricholepis*, *S*. *spec*. *nov*. *(‘mongomoensis’)* resembles *S*. *melanotricha*, *S*. *spec*. *nov*. *(‘pedicellata’)* resembles *S*. *afroreflexa* and *S*. *spec*. *nov*. *(‘pseudohispidior’)* superficially resembles *S*. *hispidior*. Comparison of these species is made under ‘Taxonomic Treatment’.

These new species are fully described and illustrated under ‘Taxonomic treatment’.

## Discussion

*Scleria* subgen. *Hypoporum* forms a well-supported monophyletic clade as was already shown before [1]. The earliest diverging lineage is always formed by the pantropical species, *S*. *lithosperma*, which conforms to *S*. sect. *Lithospermae* (C.B.Clarke) C.B.Clarke as described by Clarke [23] (Figs 2, 3 and 4). Remarkably, amplification for the ITS marker failed several times for this species, as was already the case in Bauters et al. [1]. Fresh, silica-dried and herbarium material was used and different primers were tested [1] but no usable sequences were obtained. A second clade, sister to all other species of *S*. subgen. *Hypoporum*, is the clade including *S*. *castanea* and *S*. *virgata* (Figs 2, 3 and 4), which is described as a new section below. The largest or core *S*. subg. *Hypoporum* clade, which can be indicated as *Scleria* sect. *Hypoporum* since it includes the lectotype species of *S*. subgen. *Hypoporum*, i.e. *S*. *pergracilis* (Nees) Kunth (designated by Kern 1961: 196 [6]), contains three to five clades depending on the data used. Phylogenetic relationships in this section are poorly resolved, however, several new species were revealed and some of the long-standing issues were finally resolved. Below, each section is discussed in more detail.

### *Scleria* sect. *Lithospermae* (C.B.Clarke) C.B.Clarke

Type: Scleria lithosperma (L.) Sw.

Diagnosis: Rhizomatous perennial. Culms often clustered, 30––80 cm long. Leaves 10–35 cm long, 1–5 mm wide, glabrous or sparsely pilose on adaxial surface; sheaths finely pilose or nearly glabrous. Inflorescence terminal *and* lateral, poorly paniculate to laxly glomerate-spicate with 1–2(–3) more or less well developed axillary spicate(–glomerate) side branches, of 1–4 distant interrupted spikes, few flowered. Spikelets 4–5.5 mm long. Glumes glabrous. Nutlet 2–2.5 mm long, oblong or ovate-elliptic, surface smooth to transversely rugulose. Species included in this section: *S*. *lithosperma* var. *lithosperma* and *S*. *lithosperma* var. *linearis*.

This monotypic section is recovered in all analyses except for the ITS analyses since multiple attempts failed to amplify a sequence for *S*. *lithosperma*. The only species in this section, *S*. *lithosperma*, is found in tropical Africa, America, Asia and Oceania. It is most frequently encountered in central America and the West Indies, but collections from Africa are not rare (e.g. [4]). Throughout Asia and Australia, a heterotypic variety also occurs, *S*. *lithosperma* var. *linearis* Benth. This variety could not be included in this study due to lack of material. While in *S*. *lithosperma* var. *lithosperma* the nutlet is always smooth (except for the rugulose depressions at the base), in *S*. *lithosperma* var. *linearis* the nutlet is always rugulose over the whole surface. This species differs from other *S*. subgen. *Hypoporum* species by its inflorescence consisting of terminal and lateral panicles (also found in *S*. *bequaertii*, *S*. *laxiflora* and *S*. *procumbens*, although these species are not closely related to *S*. *lithosperma*) and the absence of spikelets clustered in glomerules. This is the most widely spread species of the genus and is represented in the coastal flora of almost all tropical regions of both hemispheres [4].

### *Scleria* sect. *Virgatae* Bauters sect. nov.

Type: *Scleria virgata* (Nees) Steud.

Rhizomatous perennials, this rhizome often woody and knotted. Large plants with culms varying between 30 and 60 cm. Leaves 20–60 cm long glabrous or pubescent. Inflorescence generally paniculate and virgately branched. Spikelets androgynous and/or staminate, solitary or clustered in glomerules. Nutlets 1.5–3.5 mm long, subglobose to oblong ovoid, often trigonous, shining, smooth (*S*. *castanea*) to tuberculate-rugose.

Species included in this section: S. castanea, S. chasmena S. didina, S. variegata and S. virgata.

This is a well-supported clade, represented in our phylogeny by *S*. *castanea* and *S*. *virgata*, and was given its own sectional name in this paper. Recently, Mayedo and Thomas [22] described two new species, *S*. *chasmena* W.Bonet Mayedo & W.W.Thomas and *S*. *didina* W.Bonet Mayedo & W.W.Thomas, clearly related to *S*. *virgata*. Based on morphology, *S*. *variegata* (Nees) Steud. also belongs to this section. Species from this section are characterised by their rather woody, often noded rhizome; their virgately branched inflorescence and their tuberculate to rugose nutlet (smooth in *S*. *castanea*). The placement of *S*. *castanea*, a species with densely clustered spikelets and smooth nutlets, in this section is rather unexpected. All species of this section are restricted to Central and South America.

### *Scleria* sect. *Hypoporum* (Nees) C.B.Clarke

**Type**: Scleria pergracilis (Nees) Kunth

**Diagnosis**: *Scleria* subgen. *Hypoporum* can be recognized by its annual or perennial habit, androgynous spikelets, a reduced hypogynium. The inflorescence is a linear spike of distant, sessile spikelet clusters subtended by a short glume-like or foliate bract. This inflorescence is mostly terminal In some cases a few lateral branches arise from the highest axil or from one of the lower axils.

**Species included in this section**: S. spec. nov. (‘ankaratrensis’), S. afroreflexa, S. andringitrensis, S. angustifolia, S. aromatica, S. bequaertii, S. bourgeaui, S. bradei, S. bulbifera, S. burchellii, S. calcicola, S. catophylla, S. cheekii, S. composita, S. cuyabensis, S. delicatula, S. distans var. distans, S. distans var. chondrocarpa, S. dregeana, S. erythrorrhiza, S. flexuosa, S. fulvipilosa, S. glabra, S. glomerulata, S. guineensis, S. hilsenbergii, S. hirtella, S. hispidior, S. hispidula, S. interrupta, S. laxiflora, S. leptostachya, S. spec. nov. (‘liberica’), S. longispiculata, S. spec. nov. (‘maypurensis’), S. melanotricha, S. spec. nov. (‘mongomoensis’), S. monticola, S. pantadenia, S. paupercula, S. spec. nov. (‘pedicellata), S. pergracilis var. pergracilis, S. pergracilis var. brachystachys, S. perpusilla, S. polyrrhiza, S. pooides, S. procumbens, S. spec. nov. (‘pseudohispidior’), S. pulchella, S. purdiei, S. pusilla, S. rehmannii, S. remota, S. richardsiae, S. robinsoniana var. robinsoniana, S. robinsoniana var. acanthocarpa, S. schliebenii, S. sheilae, S. sobolifer, S. spicata, S. suaveolens, S. tenella, S. tricholepis, S. tricristata, S. verticillata, S. verseyfitzgeraldii, S. welwitschii, S. woodii and S. zambesica.

This section contains all species with a characteristic Hypoporum morphology, mainly recognisable by their Hypoporum (s.s. or s.l.) inflorescence type [1] in which spikelets are clustered in glomerules. Depending on the marker(s) used, the resulting phylogenies were differing. In the ITS analyses, four different clades were recovered, while in the ETS analyses, Clade III was split in IIIa and IIIb. In the *ndhF* analyses, four clades were also recovered, but here some of the species of Clade II (nuclear analyses) are now included in Clade III. In the *rps16* analyses, Clade I, III and IV were recovered, while Clade II species were again scattered among the other clades. In the *BEAST analysis, only three clades are recovered with Clade II and III merged into one clade. In all analyses performed for this study, relationships between the clades of *S*. sect. *Hypoporum* are not resolved. A large polytomy is found as the backbone of this section.

*Clade A–The black haired Hypoporum species*. (This clade corresponds with Clade I from the nuclear and chloroplast analyses). All species from this clade have rather remarkable reddish-black hairs on their glumes. The glumes are often very densely covered by these hairs (e.g. *Scleria catophylla*). The glomerate-spicate inflorescences of these species are never branched. Species from this clade are all from Africa, except *S*. *distans* which is also found in the Americas. *Scleria distans* from the Americas invariably has smooth nutlets, while in Africa some specimens have tuberculated nutlets and these represent the variety *S*. *distans* var. *chondrocarpa* (Nelmes) Lye. Immature specimens of *S*. *distans* often have whitish hairs instead of the reddish-black ones. In the *BEAST analysis, *S*. *tricholepis* is included in this clade. This species is widely distributed throughout Africa according to Nelmes [9] but the identity of the specimen in this study is uncertain. This specimen (Drummond & Rutherfort-Smith 7438) was identified as *S*. *hirtella* by Robinson [4, 8]. However, our results indicate that this specimen is not related to *S*. *hirtella*. Morphologically, it shows the most resemblance with type material of *S*. *tricholepis*. Many specimens of *S*. *tricholepis* cited by Nelmes [9] were studied, and a remarkable variability in these specimens was found. Either *S*. *tricholepis* is a very variable species, or it might represent a species complex that needs to be fragmented in several species. We tried to include additional accessions from this species in the molecular study, but amplification failed multiple times. Until we can resolve this taxonomic issue, we treat the specimen included here as *S*. *tricholepis*.

*Clade B*. (In the *BEAST analysis this is the equivalent of Clade II and III from the nuclear and chloroplast analyses). This clade is not equally resolved in the different analyses. All analyses, however, do recover a clade with three American species (*S*. *hirtella*, *S*. *interrupta* and *S*. *verticillata*) and two African species (*S*. *richardsiae* and *S*. *sobolifer*). All too often, the name *S*. *verticillata* has been used for specimens collected in Central and South America. Our results place these collections scattered among non-related species (e.g. *S*. *burchellii* C.B.Clarke, *S*. *tenella*, *S*. *spec*. *nov*. *(‘maypurensis’)*; all of these specimens were collected under the name *S*. *verticillata*). Although identified as ‘verticillata’ because they look very similar morphologically, they are not closely related. A high degree of morphological similarity between species is one of the major issues in *S*. subgen. *Hypoporum*. Very few morphological characters enable to distinguish species and convergence has occurred on many occasions. Our molecular phylogenetic results establish that *S*. *verticillata* is restricted North America. and cannot be confused with any other species from that region. *Scleria verticillata* is easily recognised by its tiny, solitary or tufted habit and its small nutlets which are trigonous-globose with a transversely-tuberuclate surface with quadrate ridges. Remarkable is the relatedness between the three American species (*S*. *hirtella*, *S*. *interrupta* and *S*. *verticillata*) and *S*. *richardsiae* (a Zambian endemic) and *S*. *sobolifer* (endemic to South Africa). This relationship postulates a separate transatlantic dispersal event. The ITS and ETS trees also place a clade with three new species, two from West Tropical Africa, i.e. *S*. *spec*. *nov*. *(‘liberica’)*, *S*. *spec*. *nov*. *(‘pedicellata’)*, and one from East Africa, i.e. *S*. *spec*. *nov*. *(‘pseudohispidior’)*, in Clade B. Another West African species new to science (*S*. *spec*. *nov*. *(‘mongomoensis)*) was excluded from the *BEAST analysis, but was also found in this clade in the nuclear analyses. *Scleria spec*. *nov*. *(‘liberica’)* was collected under the name *S*. *hirtella* or *S*. *interrupta*. However, our molecular results indicate that these species only occur on the American continent. *Scleria spec*. *nov*. *(‘mongomoensis’)* was collected as *S*. *melanotricha* with which it shares many morphological features. *Scleria spec*. *nov*. *(‘pedicellata’)* looks somewhat similar to *S*. *melanotricha* and *S*. *afroreflexa* Lye, but this similarity is only superficial. *Scleria spec*. *nov*. *(‘peudohispidior’)* was only once collected as *S*. *hispidior*. Although the species key of Nelmes [9] indeed leads to *S*. *hispidior*, the newly described species does not look similar at all. The four new species mentioned above are described under ‘Taxonomic Treatment’. These results show a clear need for a thorough revision of the *Scleria* species from West Tropical Africa.

Next, two clades corresponding with Clade III (a & b) are found in the polytomy of Clade B. Both clades are sometimes recovered as monophyletic (as IIIa,b in Figs 2 and 3) while in other analyses it is separated in Clade IIIa and IIIb (S3 and S4 Figs, only for ETS analyses). First, two African species *Scleria flexuosa* and *S*. *pulchella* are always recovered as sister species. *Scleria flexuosa* produces slender subterranean stolons ending in a fleshy tuber. This tuber is only recovered by careful collection and confusion is often raised by specimens of this species that were collected without the fleshy tuber. All analyses confirmed the relatedness of *S*. *flexuosa* with *S*. *pulchella*, a very small species, rarely collected and actually only known from its type locality in Angola. There has been a lot of confusion with *S*. *suaveolens* Nelmes since this species looks almost exactly the same as *S*. *pulchella*. The species *S*. *suaveolens* was described by Nelmes [9] but not recognised by most authors (e.g. [4]) who synonymised it with *S*. *pulchella*. However, our results show that both species are not closely related. Morphologically *S*. *suaveolens* is generally larger with shortly branched inflorescences, while *S*. *pulchella* normally has unbranched inflorescences. The second clade corresponding with Clade III is a well-supported clade with 11 species, also recovered in the polytomy of Clade B. *Scleria bequaertii*, *S*. *erythrorrhiza*, *S*. *laxiflora* and *S*. *longispiculata* are clearly related by their shared morphological features. All have a hypogynium-like structure at the base of the nutlet, a feature normally not present in *S*. subgen. *Hypoporum*. In *S*. *bequaertii* and *S*. *erythrorrhiza*, this structure is somewhat spongy and fleshy, up to 1 mm long, as is the case in *S*. *procumbens*, a clearly related species not included in this study. Whether this structure truly is homologous to the hypogynium found in other *Scleria* has not yet been proven, however, its resemblance is remarkable. A character also connecting *S*. *bulbifera* and *S*. *schliebenii* to the above mentioned species of Clade B, is the stem which is distinctly reddish in the upper half of the internodia. This character is rather subtle, but consistent in all studied collections. *Scleria bulbifera* is a widely distributed species and the name is used for all collections with a bulbous stem-base. In our analyses, two groups were always recovered, a group of *S*. *bulbifera* with the bulbs attached to long stolons (as in the type material) and a group of species with the bulbs directly attached to each other. When studying the type material of the long list of synonyms of *S*. *bulbifera* we found several specimens where the bulbs were firmly attached forming a rhizome-like structure. The oldest published name representing such a specimen is *S*. *schliebenii*, a name we now accept for the rhizomatous species. Also, their nutlet is distinctive: the mature nutlets of *S*. *bulbifera* are most often reticulated, while nutlets in *S*. *schliebenii* have a more or less smooth surface.

The only American representative of Clade B is *Scleria spicata*, a species morphologically clearly similar to *S*. *welwitschii* (e.g. [4]). This relationship is confirmed in this study. Both species have very long inflorescences with long, lax and more or less drooping branches [4]. Robinson [4] wondered if these two species should be merged under one name since their only difference is a swollen stem base in *S*. *spicata* (a character occasionally also found in *S*. *welwitschii*, e.g. Welwitsch 7138 (K!)). *Scleria rehmannii*, a species that is somewhat similar in habit is also included in Clade B. This species is very common in its distribution area and can be dominant in e.g. dambos in Zambia (pers. observation). Many collections of this species are known to have long spikelets, up to 2 cm, with distichously placed empty glumes. It is not yet known if this is an infection or something else but no fertile plants with deformed spikelets were observed in the field and herbaria. Closely related to *S*. *rehmannii* is the very rare *S*. *fulvipilosa*, one of those species collected only (to the authors knowledge) by E.A. Robinson. Robinson [4] remarked that this endemic of Zambia is very sensitive to fire and he had observed populations suffering badly after fires. This species is easily recognised by its hairy fruits, a feature which is unique in *S*. subgen. *Hypoporum*. Finally, also a species new to science, *S*. *spec*. *nov*. *(‘ankaratrensis’)*, endemic to Madagascar, is included in this clade. This species is described under ‘Taxonomic Treatment’.

*Clade C*. This clade is resolved into a large polytomy only separating two species at the base: *Scleria tricristata* and *S*. *delicatula*, two species clearly related by the protruding, translucent tissue on the nutlets [44] Next, *S*. *zambesica* is on a separate branch, this again is one of Robinson’s gatherings. All other species in Clade IV are in a polytomy. However, some relationships can be uncovered. *Sclera pergracilis* was always split into two clades, one of this clades corresponding with the *S*. *pergracilis* var. *brachystachys* described by Nelmes [9]. *Scleria pooides* Ridl., a slender branching species is related to the morphologically rather similar *S*. *robinsoniana* J.Raynal. Also, the separation between *S*. *woodii* C.B.Clarke and *S*. *polyrrhiza* E.A.Robinson is not very clear. Morphologically they can be separated as *S*. *woodii* only has one rhizome each year, while *S*. *polyrrhiza* produces multiple rhizomes simultaneously. However, based on the molecular data their relationship seems to be more complex. Here, we consider them to be two distinct species, but a thorough study of this species complex may reveal intriguing relationships within *S*. *woodii* as well between both species. An all American clade is recovered in almost all analyses: *S*. *composita*, *S*. *leptostachya*, *S*. *pusilla* and *S*. *tenella*. Both *S*. *pusilla* and *S*. *tenella* are often confused with *S*. *verticillata* because of their similar morphological habit. Initially, both species were included in our analyses under the name *S*. *verticillata*, but our results indicated these as different taxa. By carefully studying the type material of all *S*. subgen. *Hypoporum* species [45], it was possible to separate several species from the *S*. *verticillata*-complex.

A species represented by two samples from Angola (SC008 and SC126) is treated as unknown in our study. In a previous study [1], we identified this species as *Scleria angustifolia*, however, after including multiple accessions for *S*. *angustifolia*, it became clear that the samples from Angola do represent a different species. It clusters with *S*. *polyrrhiza* in the *BEAST analyses but it seems morphologically different. The Angolan accessions might be *S*. *polyrrhiza*, but more likely represent new species to science. However, our molecular results are not consistent for these accessions and more material is needed before we can conclude on the status these specimens.

## Taxonomic treatment

### Scleria ankaratrensis *Bauters* sp. nov. (Figs 5 and 6A)

**Fig 5:**
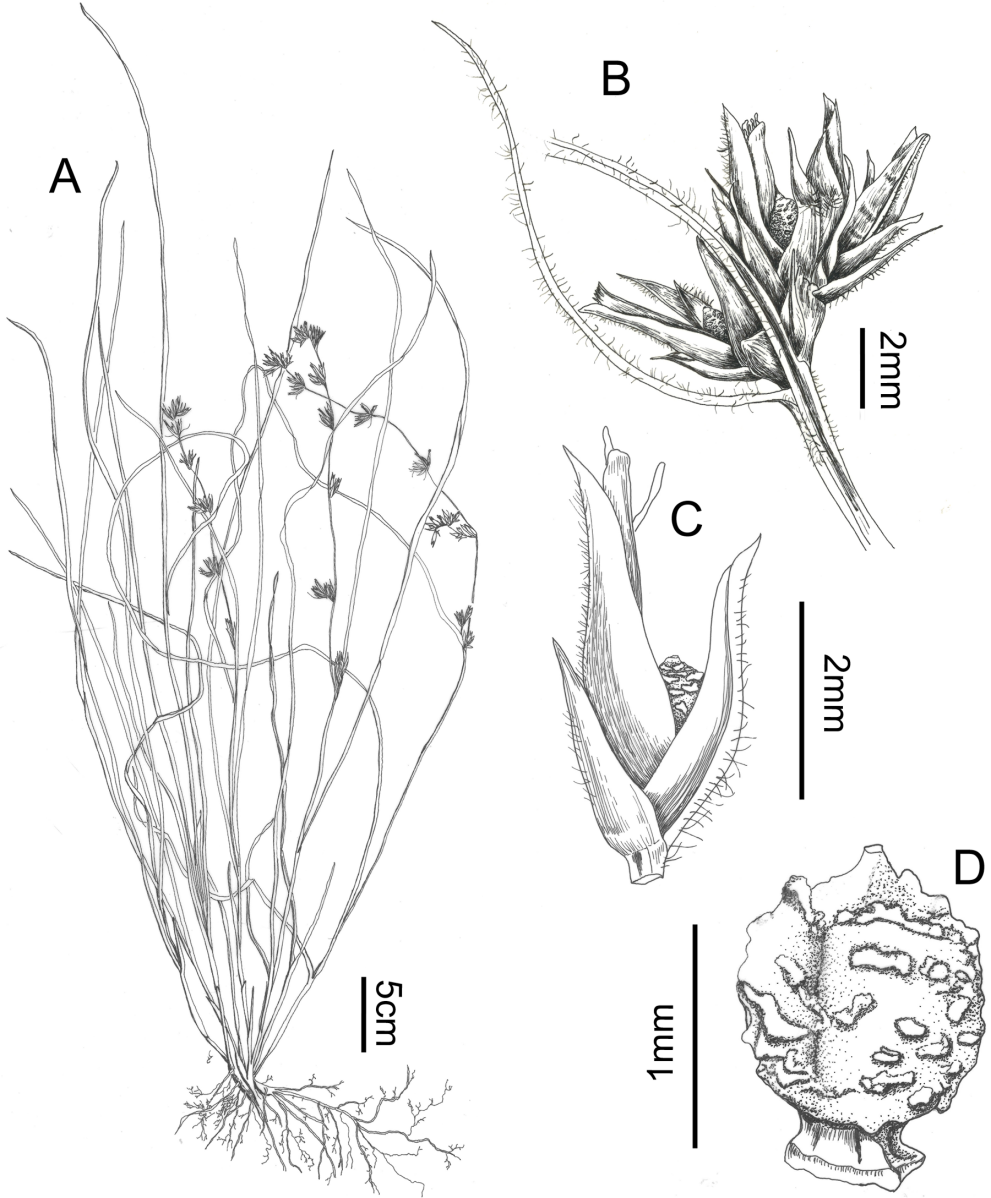
***Scleria ankaratrensis*:** A. habit, B. detail of glomerule, C. detail of a spikelet, D. Nutlet (all drawn from Larridon I. et al. 2010–0340 (GENT)).

**Fig 6:**
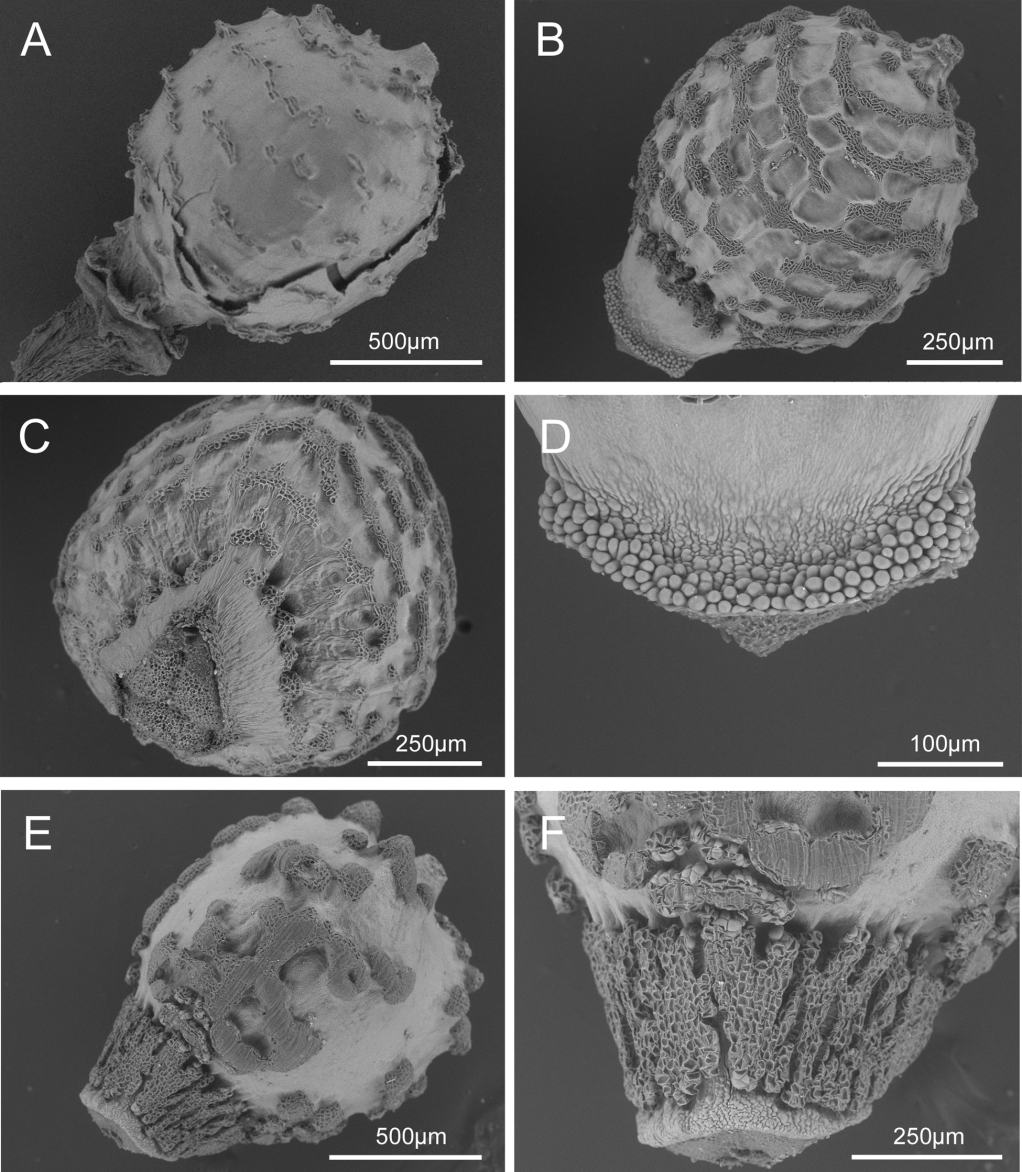
SEM micrographs of: A. nutlet of *Scleria ankaratrensis* (Larridon et al. 2010–340 (GENT)), B. nutlet of *Scleria liberica* (NBT JR53 (GENT)), C. underside of nutlet of *Scleria liberica* (NBT JR53 (GENT)), D. tuberculated rim at base of nutlet of *Scleria liberica* (NBT JR53 (GENT)), E. nutlet of *Scleria maypurensis* (Gröger A. & Garcia R. 1103 (GENT), F. details of tissue covering the stipe of *Scleria maypurensis* (Gröger A. & Garcia R. 1103 (GENT). Drawn by Judit Fehér (Ghent University).

**Scleria ankaratrensis** *Bauters* **sp. nov.** [urn:lsid:ipni.org:names:77187920–1] **Type**: Madagascar. Ankaratra, Lac Froid, 19°20’45.0”S 47°20’19.8”E, alt. 1650 m, 24 April 2010, *Larridon I*., *Huygh W*., *Reynders M*. and *Muasya A*.*M*. (with Vonona Randrianasolo) 2010–0340 (holotype: GENT!, isotypes: BOL!, K!, TAN!)

**Description**: Tufted, slightly decumbent annual herb. Roots fibrous, 0.2 mm across, purplish, rhizome absent. Culms up to 22 cm long and 0.2–0.3 mm thick at mid height, slightly triangular, with white hairs (0.2 mm long). Leaves tristichous, up to 26 cm long sometimes overtopping the culms, 1.0–1.2 mm wide, densely hairy with white hairs of more or less 0.2 mm (–0.4 mm). Sheaths glabrous or densely hairy, reddish–purplish. Ligule absent and contraligule convex and densely hairy. Inflorescence terminal glomerate-spicate, up to 9 cm long, sometimes slightly branched near the base with branches up to 1 cm long. Glomerules max. 6 along the main axis, following a prophyll branching pattern, always erect, each with (2–) 4 spikelets; glomerule bract up to 2 cm with small basal part and longer awn, hairy with white hairs. Spikelets 4–4.5 mm long, all androgynous, prophyll of the spikelets 1 mm long, often with additional spikelet in its axil. Glumes 5–many, reddish brown with green midrib; lowest glume (empty) 2.2 mm long, mucro 0.2 mm; second glume (bearing pistillate flower) 2.7 mm long, mucro 0.2 mm; third glume 3.9 mm long, mucro 0.1 mm long, these lower glumes hairy on the midrib; higher (all staminate) glumes, few to many, 4 mm or smaller, spirally arranged; staminate glumes bearing 0–2 stamens. Flowers unisexual, style trifid dark brown coloured circa 2.6 mm long of which branches 1.4 mm; stamens 2, filaments ca. 4 mm long, anthers ca. 2 mm with apiculate connective of 0.1 mm. Nutlet 1.3–1.6 mm long, 0.8 mm wide, globose–ovoid, surface slightly trabeculate, apex truncate, short (0.1 mm) and base trigonous, darker in colour; hypogynium absent.

**Habitat**: On boggy lake edges with pines, *Drosera* and *Utricularia*.

**Distribution**: Currently only known from its type locality

**Etymology**: The name ‘ankaratrensis’ refers to the volcanic Ankaratra area in Madagascar, where this species was discovered.

**Discussion**: This species is recognised by a decumbent annual habit, a branched inflorescence with simple branches, hairy glumes and a trabeculate nutlet. The glumes are strongly hairy along the midrib. It is a member of Clade II of *Scleria* sect. *Hypoporum* and most closely related to *S*. *fulvipilosa*, *S*. *rehmannii*, *S*. *spicata* and *S*. *welwitschii*. Morphologically, it is not similar to any of those species. We compare it with *S*. *hirtella* and *S*. *tricholepis* in Table 2, although these species have a different distribution area, they have a slightly similar morphology.

**Table 2:** Comparisson between *S*. *ankaratrensis*, *S*. *hirtella* and *S*. *tricholepis*.

Character	*S*. *ankaratrensis*	*S*. *hirtella*	*S*. *tricholepis*
Habit	tufted, decumbent annual	tufted, slender annual	tufted annual
Culms	up to 22 cm tall × 0.2–0.3 mm thick	up to 60 cm tall × 1.5 mm thick	up to 55 cm long × 0.3–1 mm thick
Leaves	up to 26 cm × 1.0–1.2 mm wide, densely hairy; contraligule convex and densely hairy	up to 15 cm long × 2–3 mm wide, glabrous or pubescent; contraligule indistinct with a fringe of hairs	up to 55 cm long or longer × 1–3 mm wide; glabrous to hairy; sheaths mostly glabrous but sometimes villous, especially at the truncated contraligule
Inflorescence	up to 9 cm long, slightly branched mostly in lower part; branches up to 1 cm	up to 35 cm long, unbranched	up to 13 cm, unbranched
Glomerules	max. 6 on main axis; each with (2–)4 spikelets	9–13, each with 3–7 spikelets	4–15, each with 3–6 spikelets
Spikelet	4–4.5 mm long	2.6–3.5 mm long	3–4 mm long
Glumes	lower glumes hairy along the midrib	lower (pistillate) glumes beset with hyaline hairs all over the surface	all glumes with withish hairs all over the surface
Nutlets	1.3–1.6 × 0.8 mm, globose-ovoid, surface slightly trabeculate	1.6–1.8 × 0.9–1.4 mm, globose to subglobose, surface smooth or sparsely tuberculate	1.25–1.6 × 1.1–1.3 mm, trigonous on cross-section, oblong-ovoid (oblong-globose), surface undulate-trabeculate to lacunose
Distribution	Madagascar	Central and South America, West Indies	Tropical Africa

### Scleria liberica *Bauters* spec. nov. (Figs 6B–6D and 7)

**Fig 7:**
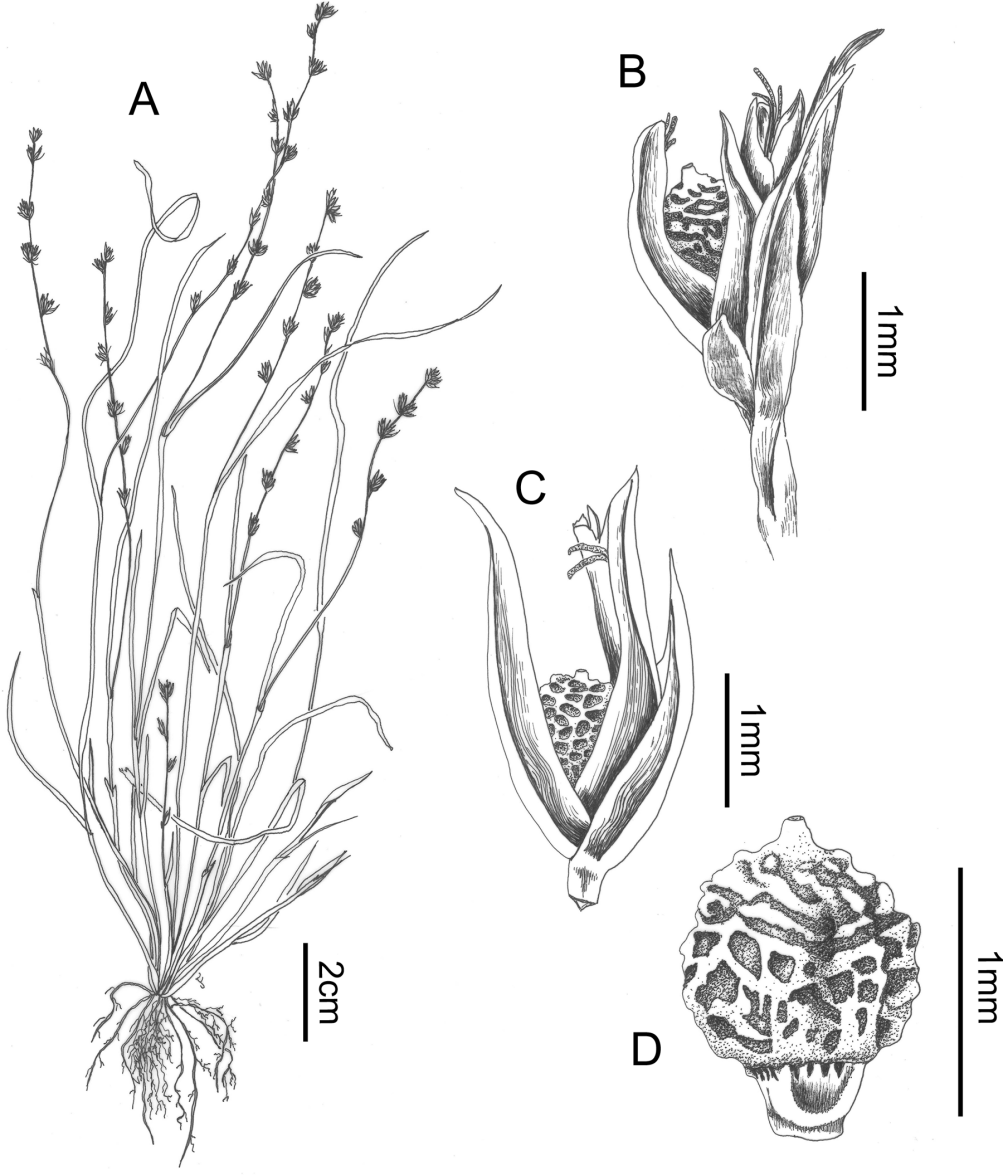
Scleria liberica. A. Habit, B. Detail of glomerule, C. Detail of spikelet, D. Nutlet (A drawn from Adam 30156 (GENT), B, C and D drawn from NBT JR053 (GENT)). Drawn by Judit Fehér (Ghent University).

**Scleria liberica** *Bauters* **spec. nov.** [urn:lsid:ipni.org:names:77187921–1] **Type:** Liberia, Grand Bassa, between Edina road and the sea. Edge of low swampy forest. 5°59.30’N, 10°11.06’W, alt. 5 m, *Jongkind C*.*C*.*H*. *with Sambolah G*. *13414* (holotype BR!; isotypes: GENT!)

**Other specimens studied:** Liberia, Yéképa, Mt. Bélé savannah, 27 september 1975, *Adam J*.*G*. *29622* (GENT); Liberia, Yéképa, savannah Bélé, 6 november 1975, *Adam J*.*G*. *30156* (GENT); Guinea, Nzérékoré, Nimba Mountains, plot JRSW01, 200 m N of pumping station. Woodland on very shallow soils and bare rock here and there, 7°40.80’N, 8°23.15’W, alt. 900 m, 21 november 2007, *Nimba Botanic Team JR53* (GENT); Guinea, Nzérékoré, Lola Prefecture, Forestiere Prov. Nimba Mountains, SMFG iron mining concession, near Zougué pump station, seeping slope of rocks and short grasses and sedges, 07°40’46”N, 08°23’06”W, alt. 1005 m, 02 october 2011, Mcpherson G. with Serein J.-Y. 21503 (BR, MO); Guinea, Région de Macenta. Dans les sites rocaillex humides sur la pente d’une montagne près de Macenta, 18 november 1962, *Lisowski S*. *s*.*n*. (BR)

**Description:** Tufted annual with erect culms. Roots fibrous, 0.1–0.3 mm across, rhizome absent. Culms 12–26 cm long, 0.3–0.5 mm wide at mid height, glabrous or very sparsely hairy with tiny white hairs. Leaves variable in length, up to 10 cm (excluding sheaths), equal to or overtopping the inflorescence, hairy with long white hairs on the upper surface on the main veins of the upper surface, vein on the underside of the leaf not hairy, sometimes also hairy along the margins. Sheaths reddish–brown (the lower ones), pale green (the higher ones), hairy on the contraligula side, becoming more dense towards the contraligule. Ligule absent, contraligule truncated, covered with white hairs. Inflorescence terminal glomerate-spicate, 4–7.5 cm long, never branched. Glomerules 4–7, ca. 1 per cm, erect, containing each 2–5 spikelets; prophyll branching pattern, often with additional spikelet in axil of prophyll; glomerule bract 3–6.5 mm long, mucro ½ to 2/3 of this length. Spikelets 2.5–4 mm long, strictly androgynous. Prophyll 1.6–2 mm long (mucro up to 0.5 mm), 0.4–0.5 mm wide. Glumes, pistillate glumes pale with green midrib, staminate glumes dark red–brown. Lower glume (empty) 2.7 mm + 0.7 mm long mucro; second glume (pistillate) 3 mm + 0.5 mm long mucro; third glume (empty) 3 mm + 0.1 mm long mucro; higher glumes all staminate, 2–3 mm long. Flowers all unisexual, style trifid, 4–5 mm long with style branches of 2 mm long. Stamen 2–3, filament 2–3 mm long, anthers 1.1–1.5 mm long with 0.3 mm long apiculate connective. Nutlet 1.1–1.2 mm long (stipe 0.2–0.3 mm long), 0.9–1.0 mm wide, (sub)globose, trigonous in cross section, surface reticulate to trabeculate, slightly muricate on angles, black when mature with raised parts appearing lighter in colour. Mature nutlets trabeculate, younger nutlets strongly reticulate, the vertical upraised parts seem to smooth out during development of the fruit. Apex truncate, 0.1 mm long, stipe trigonous with five pores on each side. Pores surrounded by translucent tissue. Hypogynium absent.

**Habitat**: Most often found on shallow soils on rocks in the Nimba mountains. Some collections are made near sea level on the edge of low swampy forest.

**Distribution**: Currently only known from the Nimba mountains in Liberia and Guinea.

**Etymology**: The name refers to the country Liberia were most collections are made. Although some collections are known from Guinea, these are always situated nearby the Liberian border in the Nimba Mountains.

**Discussion**: Often confused with the American *S*. *interrupta*. A comparison is made in Table 3. This species is most often found on shallow soils on rocks in the Nimba mountains. Some collections are made on the edge of low swampy forest at sea level. A similar distribution pattern can be found in many annual *S*. subgen. *Hypoporum* species, e.g. *S*. *pergracilis* which can be found in shallow soils on rocky outcrops but is also often collected in wet and lower situated grasslands. Although it clusters with *S*. *pedicellata*, *S*. *pseudohispidior* and *S*. *mongomoensis* it does not have mophological affinities with these species. We compare *S*. *liberica* with *S*. *interrupta* and *S*. *tricholepis*, two morphologically similar species

**Table 3:** Comparison between *S*. *liberica*, *S*. *interrupta* and *S*. *tricholepis*.

Character	*S*. *liberica*	*S*. *interrupta*	*S*. *tricholepis*
Habit	tufted annual	loosely tufted annual	tufted annual
Culms	up to 26 cm tall × 0.3–0.5 mm thick	up to 50 cm tall × max. 1.5 mm thick	up to 55 cm long × 0.3–1 mm thick
Leaves	as long as or longer than culms; hairy with long white hairs on the vains of the upper surface, sheaths hairy on contraligule side; contraligule truncate, covered with white hairs	up to 20 cm × 1–2 mm, pubescent; sheaths pubescent; contraligule unpronounced with tuft of hairs	as long as or longer than culms × 1–3 mm wide; glabrous to hairy; sheaths mostly glabrous but sometimes hairy, especially at the truncate contraligule
Inflorescence	4–7.5 cm long, unbranched	up to 10 cm long, unbranched	up to 13 cm, unbranched
Glomerules	4–7, each with 2–5 spikelets	5–13, each with 2–6 spikelets	4–15, each with 3–6 spikelets
Spikelet	2.5–4 mm long	2–4 mm long	3–4 mm long
Glumes	glabrous or slightly hairy	with long white (0.3 mm), sometimes dark brown hairs	with withish hairs all over
Nutlets	1.1–1.2 × 0.9–1.0 mm, surface reticulate to trabeculate	1–1.5 × 1.2–1.4 mm, globose, surface rugose-verrucose or tuberculate	1.25–1.6 × 1.1–1.3 mm, oblong-ovoid (oblong-globose), surface undulate-trabeculate to lacunose
Distribution	Guinea and Liberia	Central and South America, West Indies	Tropical Africa

### Scleria maypurensis *Bauters* spec. nov. (Figs 6E, 6F and 8)

**Fig 8:**
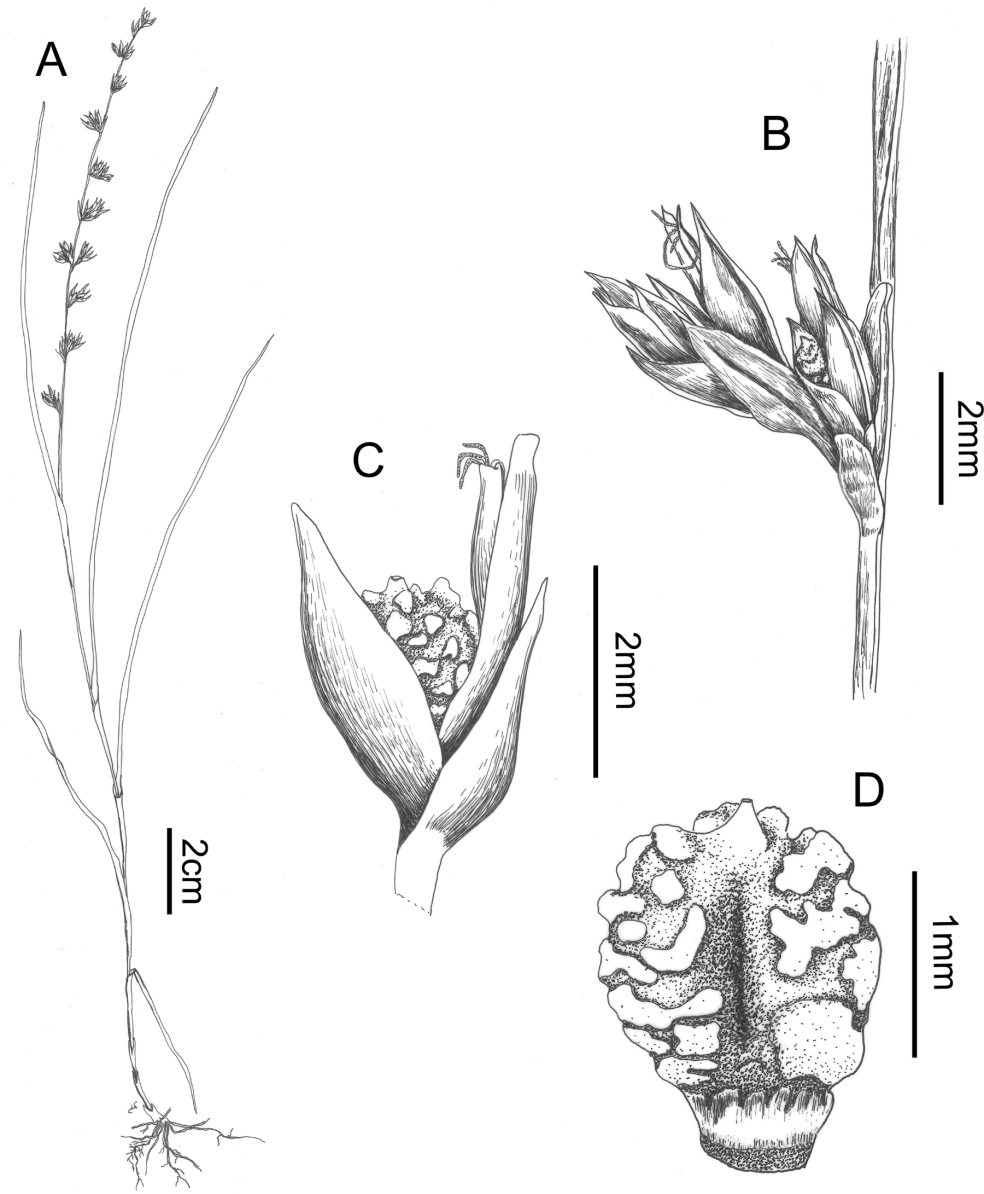
Scleria maypurensis. A. habit, B. detail of glomerule, C. detail of spikelet, D. nutlet (all drawn from Gröger A. & Garcia R. 1103 (GENT)). Drawn by Judit Fehér (Ghent University).

**Scleria maypurensis** *Bauters* **spec. nov.** [urn:lsid:ipni.org:names:77187922–1] **Type:** Venezuela, Edo. Amazonas, DPTO. Atures: carretere Pto. Ayacucho hacia Samariapo: km 42; 1 km al Sur de Guayabal al lado occidental de la carretera: Cerro Patos, 5°21’N 67°43’W en sitios humedos, entre laminas de granite, 22 September 1993, *Gröger A*. *& Garcia R*. *1103* (holotype: GENT!; isotypes: M, TFAV, VEN) (the VEN specimen might be destroyed since the herbarium in Caracas is completely abandoned (correspondence with Andreas Gröger)).

**Description:** Annual, not in tufts. Roots small, 0.3–04 mm across, purplish. Culms up to 25 cm long, 0.4–0.5 mm wide, glabrous. Leaves up to 14 cm (without sheaths), 0.7–1.2 mm wide, glabrous. Sheaths up to 3(–4.5) cm long, reddish–brown to purplish, hairy, hairs 0.2 mm long, white. Ligule absent, contraligule V–shaped, densely hairy along the edges. Inflorescence terminal glomerate-spicata, 7.5–9.5 cm long (starting from lowest glume), rarely branched in axil of lowest glomerule, these branches up to 1 cm long. Glomerules 2–10, following prophyll branching pattern, containing 2–4 spikelets, erect; glomerule bract 1 to 1.7 mm long, scaly, transparent, glabrous, rounded at the tip. Spikelets 3–4 mm long, all androgynous; prophyll of spikelets up to 2 mm long, scaly, transparent. Glumes 5 –many, very variable, lowest glume (empty), up to 4 mm long; second glume (pistillate) also up to 4 mm, higher glumes (staminate) only 3.5 mm long. Flowers unisexual, style trifid, ca. 2 mm long, branches up to 0.6 mm long; stamens 2, filaments ca. 2.5–3 mm long, anthers not seen. Nutlet 1.5 mm long, 1.0 mm wide, white with three darker stripes, subglobose (without trigonous stipe), surface roughly tuberculate, apex with small style remnant (0.1 mm), stipe trigonous, covered with translucent tissue. Hypogynium absent/obsolete.

**Habitat**: On granite outcrops between crevices in the rocks.

**Distribution**: This species in only known from its type locality. This is found in Puerto Ayacucho in the Maypures area.

**Etymology**: The name ‘maypurensis’ refers to the area where this species was discovered.

**Discussion**: This species is recognised by its small annual habit and its remarkably sculptured nutlets. It was previously confused with *Scleria verticillata* (see Table 4 for comparison with this species).

**Table 4:** Comparison between *S*. *maypurensis* and *S*. *verticillata*.

Character	*S*. *maypurensis*	*S*. *verticillata*
Habit	solitary annual	tufted annual
Culms	up to 25 cm tall, 0.4–0.5 mm wide, glabrous	up to 60 cm tall, glabrous to pubescent
Leaves	up to 18 cm long, 0.7–1.2 mm wide, glabrous	up to 30 cm long, shorter than the culms, 0.5–3.0 mm wide, glabrous to pubescent
Inflorescence	7.5–9.5 cm long, rarely branched in axil of lowest glomerule, these branches up to 1 cm long	up to 15 cm long, unbranched
Glomerules	2–10, each with 2–4 spikelets	2–9, each with 2–5 spikelets
Spikelet	3–4 mm long	2–3 mm long, appearing as if verticillate
Glumes	glabrous	glabrous
Nutlets	1.5 mm long, 1.0 mm wide, white with three darker stripes, subglobose, surface roughly tuberculate, stipe covered with translucent tissue, no pores visible	1–1.5 mm long, sometimes purplish, globose to trigonous, reticulate to verrucose, mucronate,; with 4–5 pores on each side
Distribution	Venezuela	Canada and USA

### Scleria mongomoensis *Bauters* sp. nov. (Figs 9, 10A and 10B)

**Fig 9:**
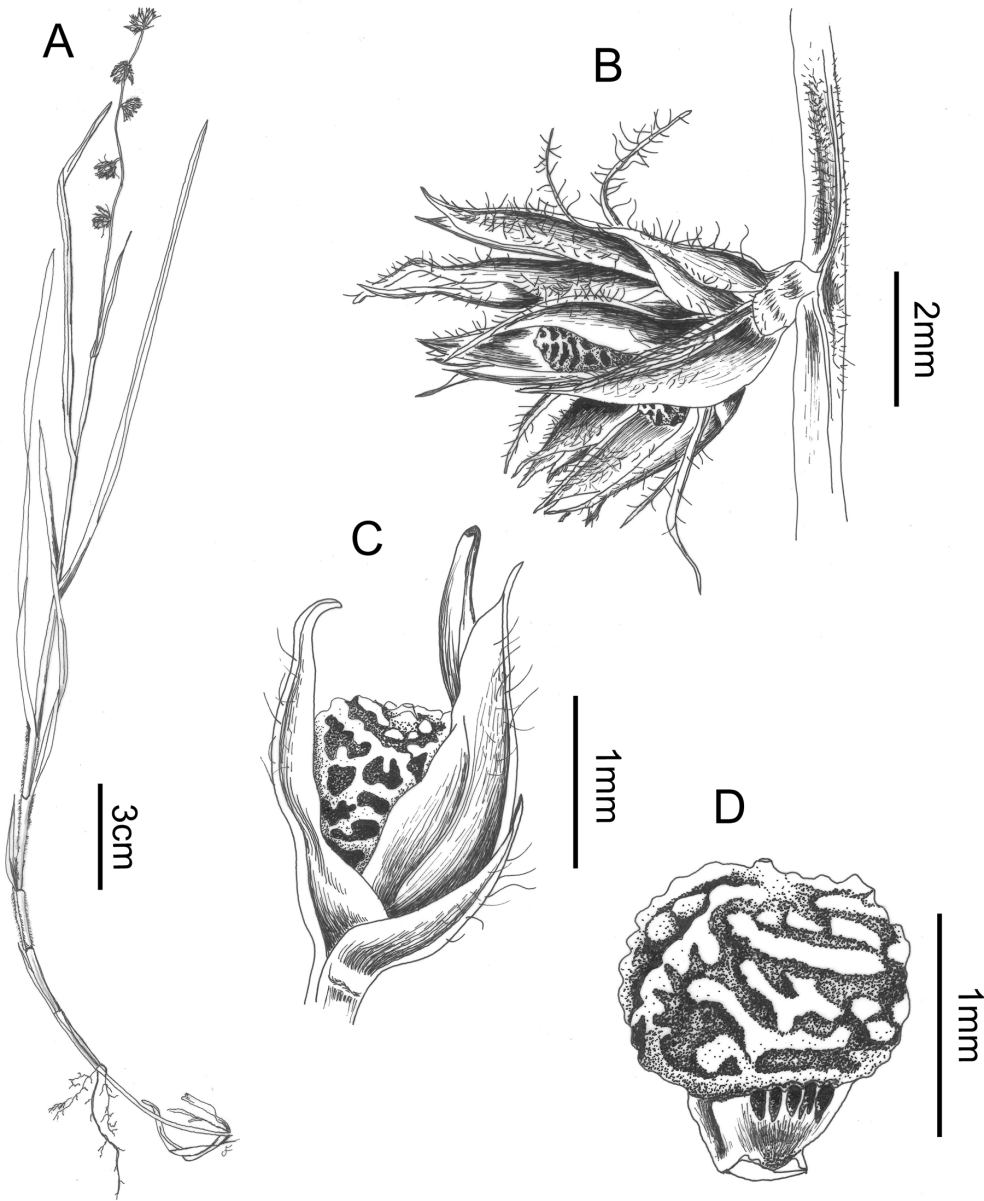
Scleria mongomoensis. A. habit, B. detail of glomerule, C. detail of spikelet, D. nutlet (drawn from Porembski S. 3609 (GENT)). Drawn by Judit Fehér (Ghent University).

**Fig 10:**
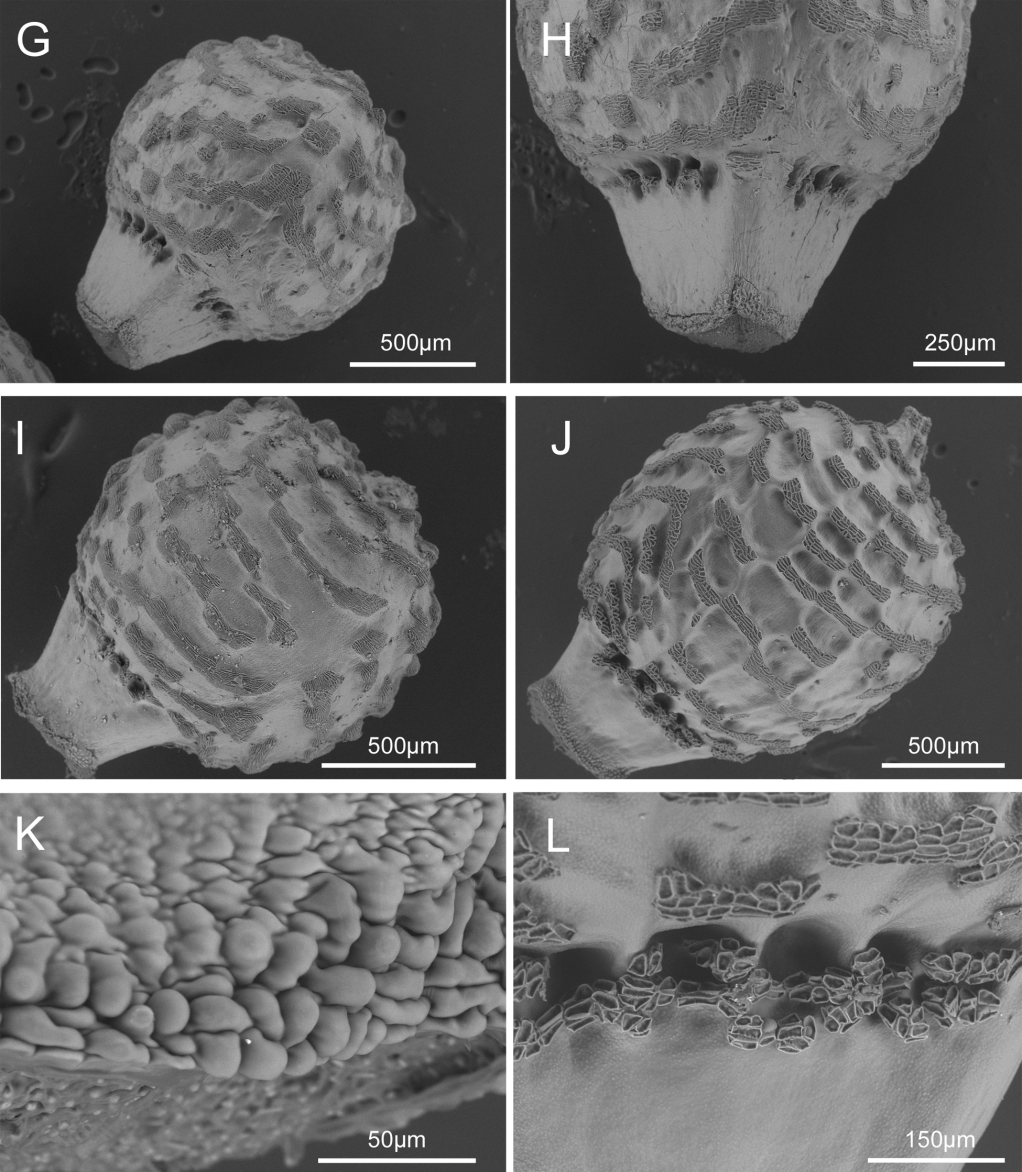
SEM micrographs of: G. nutlet of *Scleria mongomoensis* (Porembski S. 3560 (GENT)), H. stipe of *Scleria monogomoensis mongomoensis* (Porembski S. 3560 (GENT)), I. nutlet of *Scleria pedicellata* (Ngok Banak L. 1736 (MO)), J. Nutlet of *Scleria pseudohispidior* (Friis et al. 7889 (WAG)), K. detail of tuberculate rim of nutlet of *Scleria pseudohispidior* (Friis et al. 7889 (WAG)), L. detail of the pores of *Scleria pseudohispidior* (Friis et al. 7889 (WAG)).

**Scleria mongomoensis** *Bauters* **sp. nov.** [urn:lsid:ipni.org:names:77187923–1] **Type**: Equatorial Guinea, South of Mongomo, granite inselberg, rock pool edge, 1°23’N 11°20’E, alt. 660 m, 19 March 1998, *Porembski S*. *3560* (holotype: GENT!)

**Other specimens studied**: Equatorial Guinea, South of Mongomo, granite inselberg, ephermeral flush vegetation, 1°23’N 11°20’E, alt. 660 m, 19 march 1998, *Porembski S*. *3562* (GENT); Equatorial Guinea, South of Monogomo, near Domo, granite inselberg, ephemeral flush vegetation, 1°21’N 11°20’E, alt. 750 m, 19 march 1998, *Porembski S*. *3609* (GENT).

**Description**: Annual, not in tufts, sometimes decumbent, rooting on the creeping stem. Roots fibrous, 0.2–0.3 mm across, purplish, rhizome absent. Culms 20–35 (–90) cm long, 0.5–1.2 mm wide, covered with long white hairs, up to 0.5 mm long, quite dense. Leaves up to 20 cm (without sheaths), 1.5–3.0 (–3.5) mm wide, densely hairy, hairs up to 0.7 mm long. Sheaths with long white hairs, purplish (at least the lower ones). Ligule absent, contraligule straight to slightly convex, covered with long white hairs. Inflorescence terminal glomerate-spicate, up to 12 cm long (starting from lowest glume), glomerate–spicate, never branched. Glomerules 4–6 (–7), following prophyll branching pattern, containing 2–5 spikelets, reflexed or sometimes spreading, stalked with stalk of 1(–2) mm; glomerule bract up to 6 mm of which awn up to 3 mm long, awn with long white hairs. Spikelets 3–5.5 mm long, all androgynous, prophyll of spikelets 1.5–2.0 mm long, transparent. Glumes 5–many, very variable, lowest glume 1.5–2.0 mm long + 0.5–1.5 mm long mucro; second glume (pistillate) 1.5–2.5 mm long + 0.5–1.5 mm long mucro; third glume 2.5–3.2 mm long+ 0.1–1.5 mm long mucro; first three glumes densely hairy, often with blackish hairs; higher glumes, all staminate, less hairy or glabrous, up to 4 mm long, decreasing in length the higher upon the axis, spirally arranged. Flowers unisexual, style trifid, ca. 4 mm long, branches up to 2.5 mm long; stamens 2, filaments ca. 4 mm, anthers 1–1.5 mm long with small connective. Nutlet 1.4–1.5 mm long, 1.3–1.5 mm wide, greyish, subglobose, trigonous from above, with three ‘oval lobes’, surface tuberculate-trabeculate, apex truncate, very flat, no protruding part, stipe trigonous with 12 pores (4 on each side), small brown rim of small tubercles. Hypogynium absent/obsolete.

**Habitat**: On granite inselbergs, on shallow soil.

**Distribution**: This species in known from a location South of Mongomo in Equatorial Guinea.

**Etymology**: The species epithet ‘mongomoensis’ refers to the type locality of this species.

**Discussion**: This species is easily confused with *Scleria melanotricha* (comparison in Table 5). It was collected as *S*. *melanotricha* on inselbergs in Equatorial Guinea. However, it can be recognised by its more decumbent habit and the more densely hairy culms and leaves. Hairs on culms and leaves are longer (up to 0.7 mm).

**Table 5:** Comparison between *S*. *melanotricha* and *S*. *mongomoensis*.

Character	*S*. *melanotricha*	*S*. *mongomoensis*
Habit	densely villous annual	densely villous annual, sometimes decumbent
Culms	up to 45(–60) cm tall, 0.5–1 mm thick, glabrous or sparsely villous	up to 35(–90) cm tall, 0.5–1.2 mm thick, covered with long white hairs (up to 0.5 mm)
Leaves	1–2 mm wide, usually subdensely villous but sometimes sparsely so; contraligule truncate, hairy	1.5–3.0(–3.5) mm wide, densely hairy; contraligule straight to slightly convex, covered with long white hairs (up to 0.5 mm)
Inflorescence	3–20 cm long, spicate, rarely shortly branched in the lower part	up to 12 cm long, unbranched
Glomerules	5–17, reflexed, often very shortly stalked	4–7, reflexed, sometimes spreading, slightly stalked
Spikelet	3–5 mm long	3–5.5 mm long
Glumes	densely hairy; with blackish, bristly hairs, mainly along midrib	densely hairy; with blackish, bristly hairs, all over
Nutlets	1–1.75 mm long, 0.8–1.6 mm broad, broadly ovoid or almost globose, surface tuberculate-trabeculate, blackish with raised parts of the surface appearing whitish	1.4–1.5 mm long, 1.3–1.5 mm broad, subglobose, trigonous on cross section, with tree oval lobes, surface tuberculate-trabeculate, with 4 pores on each side
Distribution	Tropical Africa	Equatorial Guinea

### Scleria pedicellata *Bauters* spec. nov. (Figs 10I and 11)

**Fig 11:**
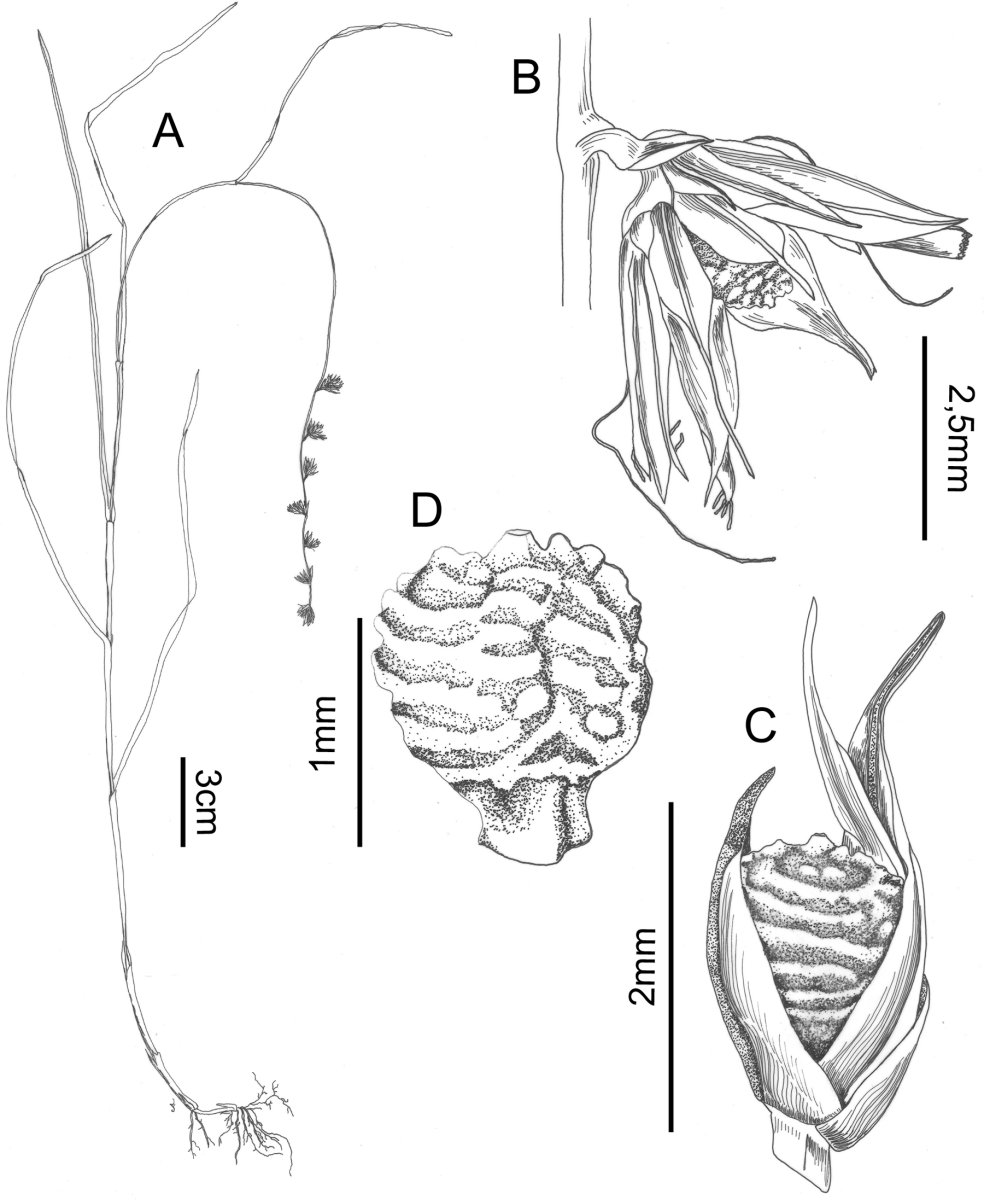
Scleria pedicellata. A. habit, B. detail of glomerule, C. detail of spikelet, D. nutlet (all drawn from Ngok Banak L. 1736). Drawn by Judit Fehér (Ghent University).

**Scleria pedicellata** *Bauters* **spec. nov.** [urn:lsid:ipni.org:names:77187924–1] **Type**: Gabon, Ogooué-ivindo, Mt. Sassamongo, rocky plateau W of Sassamongo village, 0°49.71’N 13°28.06’E, Alt. 485m, 14 mai 2003, *Ngok Banak L*. *1736* (holotype: MO!; isotypes: BRLU!, LBV, P!, WAG!)

**Other specimens studied:** Gabon, Ogooué-ivindo, Parc National de l’invindo, Camp du Bai de Langoué, 0°11.20’S 12°33.40’E, Alt. 461m, 15 march 2004, *Ngok Banak L*. *1956* (BR!, WAG!); Gabon, Ogooué-ivindo, Parc National de l’invindo, Camp du Bai de Langoué, 0°11.20’S 12°33.40’E, Alt. 461m, 15 march 2004, *Ngok Banak L*. *1965* (WAG!); Gabon, Woleu-Ntem, Minkébé National Parc, southern inselberg area, 1°22.21’N 12°32.27°E, Alt. 686m, 4 mai 2003, *Ngok Banak L*. *1613* (WAG).

**Description**: Loosely tufted, slightly decumbent annual herb. Roots fibrous, 0.1 mm across, yellowish–brownish, rhizome absent. Culms 35–50 cm long, 0.4–0.5 mm thick at mid height, not erect but slightly scrambling, glabrous. Leaves tristichous, up to 17 cm long, 1.5–3.5 mm wide, glabrous. Sheaths glabrous, reddish–purplish (at least the lower ones). Ligule absent, contraligule convex, with very few hairs. Inflorescence terminal glomerate-spicate, up to 10 cm long, unbranched but the stalked glomerules occassionaly do form branches of 2.5 mm long. Glomerules 3–7, following a prophyll branching patterns, containing 3–4 spikelets, always reflexed, pedicellate, with stalks up to 2.5 mm long; glomerule bract up to 5 mm long (1.5 base + 2.5 mm awn or longer), glabrous. Spikelets 4–5 mm long, androgynous but sometimes subandrogynous, then spikelets shorter, prophyll of the spikelets up to 3 mm, often with additional spikelet in its axil. Glumes 4–many, dark reddish brown, lighter near the edges, green midrib; lowest glume 1.5–2.0 mm long + 1.3–1.5 mm long mucro; second glume (pistillate) 1.8–2.0 mm long + 1.2 mm long mucro; third glume 3.5 + 0.1 mm long mucro; higher glumes (staminate), spirally arranged, 1–many, 3–4 mm long, decreasing in length with height, most bearing 2 stamens. Flowers unisexual, style trifid, dark brown coloured ca. (1–)2 mm long, style branches ca. 1.5 mm long; stamens 2, filaments 3.0–3.2 mm long, anthers 1.8 mm long with 0.1 mm long connective. Nutlet 1.2–1.5 mm long (stipe included, 0.4 mm long), 1.2–1.3 mm wide, greyish, more or less globose, nutlet surface trabeculate, apex truncate, short (0.2 mm) and base trigonous; hypogynium absent.

**Habitat**: Found on rocky plateau with dry to humid prairies.

**Distribution**: Currently only known from Ogooué-ivindo and Woleu-Ntem provinces, most likely also present in regions in between these provinces.

**Etymology**: The name ‘pedicellata’ refers to the stalked glomerules. These glomerules are drooping.

**Discussion**: This species is recognised by its annual, tufted habit with scrambling stems, pedicellated, reflexed glomerules and a globose, trabeculated nutlet. The stalked, pendulent glomerules resemble *S*. *afroreflexa*, with which it is compared in Table 6.

**Table 6:** Comparison between *S*. *afroreflexa* and *S*. *pedicellata*.

Character	*S*. *afroreflexa*	*S*. *pedicellata*
Habit	delicate annual	loosely tufted, slightly decumbent annaul
Culms	10–50 cm × 0.3–0.8 mm, glabrous to sparseley hairy	35–50 cm × 0.4–0.5 mm thick, glabrous
Leaves	blades 2–9 cm long × 0.8–1.8 mm, with spreading transparent hairs at least on margin and major nerves	blades up to 17 cm long × 1.5–3.5 mm, glabrous
Inflorescence	3–9 cm long, with branches of 1–2 cm, these branches often reflexed	up to 10 cm, unbranched, although the stalked glomerules occassionaly do form branches (only 2.5 mm long)
Glomerules	5–9 on the main axis, branches with 1–3 glomerules, each containing 2–10 spikelets, always reflexed	3–7, each containing 3–4 spikelets, always reflexed
Spikelet	3–4 mm long	4–5 mm long
Glumes	midrib slightly scabrid	completely glabrous
Nutlets	1.0–1.1 mm × 0.7–0.8 mm, obtusely triangular with 3 oval lobes above and more sharpy triangular base, surface transversely wrinkled	1.2–1.5 mm × 1.2–1.3 mm, globose, without lobes, surface trabeculate
Distribution	Western Cameroon	Gabon

### Scleria pseudohispidior *Bauters* spec. nov. (Figs 10J–10L and 12)

**Fig 12:**
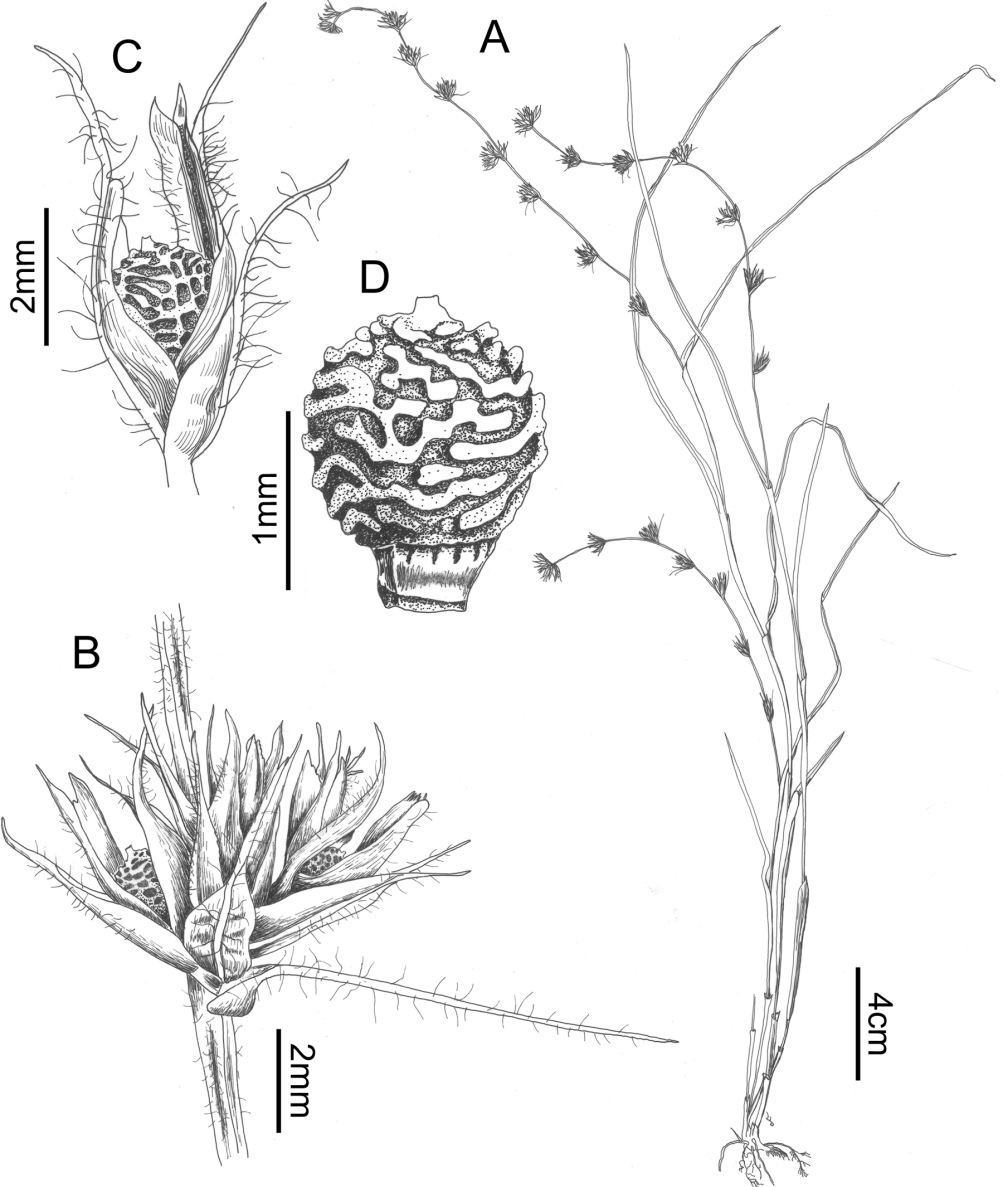
Scleria pseudohispidior. A. habit, B. detail of glomerule, C. detail of spikelet, D. nutlet (all drawn from Friis 7889 (WAG)). Drawn by Judit Fehér (Ghent University).

**Scleria pseudohispidior** *Bauters* **spec. nov.** [urn:lsid:ipni.org:names:77187925–1] **Type:** Ethiopia, Wellega Region, ca. 20 km south of Asosa, 9°55’N 34°40’E, alt. 1500 m, 23 october 1996, *Friis I*., *Bidgood S*., *Semon F*., *Jensen M*. *& Gashaw M*. *7889* (holotype: WAG!, isotypes: C, ETH, K!).

**Description:** Loosely tufted annual. Roots fibrous, 0.1–0.2 across, rhizome absent. Culms up to 55 cm long, 0.9–1.0 mm wide at mid height, erect, hairy with long white hairs, these hairs up to 0.5 mm long. Leaves tristichous, up to 25 cm long (without sheath), 1.5–3.9 mm wide, with long white hairs, 0.6–0.7 mm long, mainly on the margins. Sheaths up to 5 cm long, hairy with hairs up to 1 mm long, pale green, reddish–brown near the base. Ligule absent, contraligule inconspicuous, hairy. Inflorescence terminal glomerate-spicate, up to 17 cm long (starting from lowest glume), never branched. Glomerules (4–)9, distant, separated 1–3 cm, higher ones closer, prophyll branching pattern, erect, containing 2–4 spikelets; glomerule bract (0.5–)2–3 mm with a mucro of up to 6 mm, hairy with long white hairs, up to 0.7 mm long. Spikelets 5–6 mm long, strictly androgynous; prophyll of the spikelets 2–3 mm long, often with additional spikelet in its axil. Glumes 4–many, pale brown with green midrib, very hairy, long mostly white hairs, sometimes black or red, mainly along and on the midrib, up to 0.7 mm long. Lowest glume (empty) 2.5 mm long + 1.5 mm long mucro; second glume (pistillate) 3.5 mm long + 1.2 mm long mucro; third glume (empty) 3.5 mm long + 1 mm long mucro. Higher glumes all staminate, up to 4 mm. Flowers unisexual, style trifid, ca. 2.2 mm long, style branches 1.2 mm long.; stamens 2, filaments up to 4 mm long, anthers 2 mm long with 0.3 mm long reddish connective on top. Nutlet 1.8 mm long (+ 0.3 mm long stipe), 1.4 mm wide, whitish with the raised parts darker in colour, ovoid, surface trabeculate to reticulate, apex truncate, short (0.1–0.2 mm) and base trigonous with pores. Hypogynium absent.

**Habitat**: Open short wooded grassland with *Terminalia*, *Syzygium guineense* subsp. *macrocarpum*, etc. On rocky outcrop surrounded by dense scrubs of *Oxytenanthera*.

**Distribution**: *Scleria pseudohispidior* is only known from its type locality in Ethiopia.

**Etymology**: The epithet ‘pseudohispidior’ refers to the resemblance with *Scleria hispidior*.

**Discussion**: This species is easily recognised. It is a rather large annual with glomerules distantly separated from each other. Although several identification keys (e.g. [9]) resolve this species as *Scleria hispidior*, this species is clearly different (Table 7 comparison between *S*. *hispidior* and *S*. *pseudohispidior*).

**Table 7:** Comparison between *S*. *hispidior* and *S*. *pseudohispidior*.

Character	*S*. *hispidior*	*S*. *pseudohispidior*
Habit	slender, hairy, tufted annual	loosely tufted, hairy annual
Culms	10–30 cm long, 0.6–0.8 mm thick, glabrous to densely villous	35–55 cm long, 0.8–1 mm thick, hairy with long white hairs (up to 0.5 mm)
Leaves	1–2.25 mm wide, usually subdensely hairy with white hairs (0.2–0.3 mm)	1.5–3.9 mm wide, with long white hairs (up to 0.5 mm)
Inflorescence	3–7 cm long	up to 17 cm long
Glomerules	3–7, each containing 2–6 crowded spikelets	4–9 distant glomerules, each containing 2–4 spikelets
Spikelet	3–6 mm long	5–6 mm long
Glumes	covered with blackish hairs	covered with whitish hairs
Nutlets	1.2–1.4 mm long, 0.8–1 mm broad, trigonous-globose, surface rugose-trabeculate to muricate	1.8 mm long, 1.4 mm broad, ovoid, surface trabeculate to reticulate
Distribution	Ethiopia and Uganda	Ethiopia

## Supporting information

S1 Table Voucher information and GenBank accession numbers included in this study. Abbreviation used for analyses, taxon, vouchers, country, herbarium code, origin and GenBank accession numbers for ETS, ITS, *ndhF* and *rps16*.
(DOCX)

S1 Fig 50% majority rule consensus tree based on BI analysis of ITS. Posterior probabilities. Posterior probabilities indicated on the respective branches when equal or higher than 0.90.
(PDF)

S2 Fig Best scoring ML tree for ITS, with bootstrap values equal or higher than 75% displayed above the branches. (PDF)

S3 Fig 50% majority rule consensus tree based on BI analysis of ETS. Posterior probabilities. Posterior probabilities indicated on the respective branches when equal or higher than 0.90.
(PDF)

S4 Fig Best scoring ML tree for ETS, with bootstrap values equal or higher than 75% displayed above the branches. (PDF)

S5 Fig 50% majority rule consensus tree based on BI analysis of the concatenated nuclear dataset. Posterior probabilities. Posterior probabilities indicated on the respective branches when equal or higher than 0.90.
(PDF)

S6 Fig Best scoring ML tree for the concatenated nuclear dataset, with bootstrap values equal or higher than 75% displayed above the branches. (PDF)

S7 Fig 50% majority rule consensus tree based on BI analysis of *ndhF*. Posterior probabilities. Posterior probabilities indicated on the respective branches when equal or higher than 0.90.
(PDF)

S8 Fig Best scoring ML tree for *ndhF*, with bootstrap values equal or higher than 75% displayed above the branches. (PDF)

S9 Fig 50% majority rule consensus tree based on BI analysis of *rps16*. Posterior probabilities. Posterior probabilities indicated on the respective branches when equal or higher than 0.90.
(PDF)

S10 Fig Best scoring ML tree for *rps16*, with bootstrap values equal or higher than 75% displayed above the branches. (PDF)

S11 Fig 50% majority rule consensus tree based on BI analysis of the concatenated chloroplast dataset. Posterior probabilities. Posterior probabilities indicated on the respective branches when equal or higher than 0.90.
(PDF)

S12 Fig Best scoring ML tree for the concatenated chloroplast dataset, with bootstrap values equal or higher than 75% displayed above the branches. (PDF)

S1 Alignment ETS1f_hypoporum.fas. (FAS)

S2 Alignment ITS_hypoporum.fas. (FAS)

S3 Alignment ndhF_hypoporum.fas. (FAS)

S4 Alignment rps16 hypoporum.fas. (FAS)
