# Supplementary material for: Molecular phylogenetic study of Scleria subgenus Hypoporum (Sclerieae, Cyperoideae, Cyperaceae) reveals several species new to science
Source: PLoS One. 2018 Sep 27;13(9):e0203478. doi: 10.1371/journal.pone.0203478 (PMC6160245; doi:10.1371/journal.pone.0203478)
Supplement: S1 Table — Abbreviation used for analyses, taxon, vouchers, country, herbarium code, origin and GenBank accession numbers for ETS, ITS, ndhF and rps16. (DOCX) [file pone.0203478.s001.docx]

**Table S1.** Voucher information and GenBank accession numbers included in this study. Abbreviation used for analyses, taxon, vouchers, country, herbarium code, origin and GenBank accession numbers for ETS, ITS, *ndhF* and *rps16*. Blanks indicate no sequence.

| **Code** | **Genus** | **Species** | **Voucher** | **Country** | **Herbarium** | **ETS** | **ITS** | ***ndhF*** | ***rps16*** |
| --- | --- | --- | --- | --- | --- | --- | --- | --- | --- |
| SC001 | Scleria | lithosperma var. lithosperma | Viane R. 2241 | Thailand | GENT | MG708867 |  |  |  |
| SC002 | Scleria | lithosperma var. lithosperma | Viane R. 824 | Ivory Coast | GENT | MG708868 |  |  |  |
| SC003 | Scleria | lithosperma var. lithosperma | Stevens W.D. & Martinez S. 25260 | Guatemala | GENT | MG708869 |  | MG708634 | MG708754 |
| SC006 | Scleria | welwitschii | Bamps, Symoens, Vanden Berghen 542 | Zimbabwe | GENT | MG708870 | MG708507 | MG708635 |  |
| SC007 | Scleria | rehmannii | Hess H.&E. 51/161 | Angola | GENT | MG708871 | MG708508 | MG708636 |  |
| SC008 | Scleria | species (unknown) | Hess H.&E. 52/597 | Angola | GENT | MG708872 | LN886807 | LN886918 | LN887036 |
| SC009 | Scleria | rehmannii | Laegaard S. 15729 | Zimbabwe | GENT | MG708873 | LN886808 | LN886919 | LN887037 |
| SC011 | Scleria | dregeana | Hess H.&E. 52/468 | Angola | GENT | MG708874 |  | MG708637 |  |
| SC012 | Scleria | dregeana | Malaisse & Goetghebeur 1137 | Democratic Republic of Congo | GENT | MG708875 | MG708509 | MG708638 | MG708755 |
| SC013 | Scleria | polyrrhiza | Hess H.&E. 52/690 | Angola | GENT | MG708876 | MG708510 | MG708639 |  |
| SC014 | Scleria | woodii | Goetghebeur P. 4493 | South Africa | GENT | MG708877 | MG708511 | MG708640 | MG708756 |
| SC016 | Scleria | flexuosa | Hess H.&E. 52/142 | Angola | GENT | MG708878 | MG708512 | MG708641 | MG708757 |
| SC017 | Scleria | flexuosa | Laegaard S. 16237 | Zimbabwe | GENT | MG708879 |  | MG708642 |  |
| SC018 | Scleria | flexuosa | Hess H.&E. 52/689 | Angola | GENT | MG708880 | MG708513 | MG708643 | MG708758 |
| SC019 | Scleria | pooides | Hess H.&E. 52/281 | Angola | GENT | MG708881 | LN886809 | LN886920 | LN887038 |
| SC020 | Scleria | pooides | Hess H.&E. 51/169 | Angola | GENT | MG708882 | MG708514 | MG708644 |  |
| SC021 | Scleria | welwitschii | Hilliard O.M. & Burtt B.L. 10089 | South Africa | GENT | MG708883 | MG708515 | MG708645 |  |
| SC022 | Scleria | spec. nov. ('ankaratrensis') | Larridon et al. 2010-0340 | Madagascar | GENT | MG708884 | MG708516 | MG708646 | MG708759 |
| SC023 | Scleria | erythrorrhiza | Malaisse & Goetghebeur 871 | Democratic Republic of Congo | GENT | MG708885 | MG708517 | MG708647 | MG708760 |
| SC024 | Scleria | pulchella | Hess H.&E. 52/1745 | Angola | GENT | MG708886 | MG708518 | MG708648 | MG708761 |
| SC025 | Scleria | erythrorrhiza | Hess H.&E. 52/289 | Angola | GENT | MG708887 | LN886810 | LN886921 | LN887039 |
| SC026 | Scleria | bulbifera | Reekmans M. 4745 | Burundi | GENT | MG708888 | MG708519 |  |  |
| SC027 | Scleria | melanotricha | Porembski S. 2401 | Benin | GENT | MG708889 | MG708520 |  | MG708762 |
| SC028 | Scleria | distans var. chondrocarpa | Desfayes M. 95.17115 | Madagascar | GENT | MG708890 | MG708521 | MG708649 | MG708763 |
| SC029 | Scleria | spec. nov. ('mongomoensis') | Porembski S. 3609 | Equatorial Guinea | GENT | MG708891 | MG708522 |  |  |
| SC030 | Scleria | woodii | Reekmans M. 6780 | Burundi | GENT | MG708892 | MG708523 | MG708650 | MG708764 |
| SC031 | Scleria | distans var. chondrocarpa | Larridon et al. 2010-0229 | Madagascar | GENT | MG708893 | MG708524 | MG708651 | MG708765 |
| SC032 | Scleria | hilsenbergii | Larridon et al. 2010-0138 | Madagascar | GENT | MG708894 | LN886812 | LN886923 | LN887041 |
| SC033 | Scleria | distans var. chondrocarpa | Larridon et al. 2010-0279 | Madagascar | GENT | MG708895 | MG708525 | MG708652 | MG708766 |
| SC034 | Scleria | distans var. chondrocarpa | Larridon et al. 2010-0241 | Madagascar | GENT | MG708896 | LN886813 | LN886924 | LN887042 |
| SC035 | Scleria | bulbifera | Reekmans M. 8724 | Burundi | GENT | MG708897 | MG708526 |  |  |
| SC036 | Scleria | distans var. distans | Reekmans M. 10474 | Burundi | GENT | MG708898 | MG708527 |  |  |
| SC037 | Scleria | distans var. distans | Reekmans M. 7239 | Burundi | GENT | MG708899 | MG708528 | MG708653 | MG708767 |
| SC039 | Scleria | distans var. distans | Gonto R. & Fernandez A. 147 | Venezuela | GENT | MG708900 | MG708529 |  |  |
| SC040 | Scleria | sobolifer | Reid C. 531 | South Africa | GENT | MG708901 | LN886814 | LN886925 | LN887043 |
| SC041 | Scleria | spicata | Harley R.M. 24658 | Brazil | GENT | MG708902 | LN886815 | LN886926 | LN887044 |
| SC042 | Scleria | woodii | Dyer M.E. 753 | Nigeria | GENT | MG708903 | LN886816 | LN886927 | LN887045 |
| SC044 | Scleria | tenella | Cremers G. & Granville J.J. 13978 | French Guiana | GENT | MG708904 | MG708530 | MG708654 | MG708768 |
| SC045 | Scleria | tenella | Maas P.J.M.; Koek-N J; et al. 7211 | French Guiana | GENT | MG708905 | MG708531 | MG708655 | MG708769 |
| SC046 | Scleria | castanea | Solomon J.C. et al. 19067 | Bolivia | GENT | MG708906 | MG708532 | MG708656 | MG708770 |
| SC047 | Scleria | castanea | Beck St. G. 22851 | Bolivia | GENT | MG708907 | LN886818 | LN886929 | LN887047 |
| SC049 | Scleria | catophylla | Hess H.&E. 51/139 | Angola | GENT | MG708908 | LN886819 | LN886930 | LN887048 |
| SC050 | Scleria | catophylla | Hess H.&E. 52/505 | Angola | GENT | MG708909 | MG708533 | MG708657 | MG708771 |
| SC051 | Scleria | catophylla | Hess H.&E. 52/844 | Angola | GENT | MG708910 | MG708534 | MG708658 | MG708772 |
| SC052 | Scleria | composita | Beck St. G. 13175 | Bolivia | GENT | MG708911 |  | MG708659 | MG708773 |
| SC053 | Scleria | purdiei | Beck St. G. 13542 | Bolivia | GENT | MG708912 | LN886820 | LN886931 | LN887049 |
| SC059 | Scleria | brownii | Wilson K.L. 8098 | Australia | GENT | MG708913 | LN886823 | LN886934 | LN887052 |
| SC070 | Scleria | calcicola | Bingham 7587 | Zambia | GENT | MG708914 | MG708535 | MG708660 | MG708774 |
| SC110 | Scleria | mackaviensis | Wilson K.L. 9871 | Australia | NE | MG708915 | LN886847 | LN886958 | LN887074 |
| SC118 | Scleria | virgata | Irwin H.S. et al. 20762 | Brazil | GENT | MG708916 | LN886849 | LN886960 | LN887076 |
| SC123 | Scleria | pergracilis var. pergracilis | Laegaard S. 21176 | Burkina Faso | GENT | MG708917 | MG708536 | MG708661 | MG708775 |
| SC124 | Scleria | suaveolens | Smith P. & Chishala C. 1844 | Zambia | GENT | MG708918 | MG708537 | MG708662 | MG708776 |
| SC126 | Scleria | species (unknown) | Hess H.&E. 52/598 | Zambia | GENT | MG708919 | MG708538 | MG708663 | MG708777 |
| SC144 | Scleria | longispiculata | Hess H.&E. 52/437 | Angola | GENT | MG708920 | MG708539 | MG708664 | MG708778 |
| SC157 | Scleria | pergracilis var. pergracilis | Porembski S. 595 | Ivory Coast | GENT |  |  | MG708665 |  |
| SC169 | Scleria | woodii | Dyer M.E. 753 | Nigeria | GENT | MG708921 | MG708540 | MG708666 | MG708779 |
| SC172 | Scleria | virgata | Vanzela A.L.L. 2 | Brazil | GENT | MG708922 | MG708541 | MG708667 | MG708780 |
| SC173 | Scleria | pergracilis var. pergracilis | Madsen J.E. 5987 | Burkina Faso | GENT | MG708923 | LN886876 | LN886988 | LN887103 |
| SC177 | Scleria | distans var. distans | Bauters K. & Coenen J. 2013-007 | USA | GENT | MG708924 | LN886877 | LN886989 | LN887104 |
| SC178 | Scleria | distans var. distans | Bauters K. & Coenen J. 2013-008 | USA | GENT | MG708925 | MG708542 | MG708668 | MG708781 |
| SC182 | Scleria | lithosperma var. lithosperma | Bauters K. & Coenen J. 2013-019 | USA | GENT | MG708926 |  | LN886990 | LN887105 |
| SC183 | Scleria | lithosperma var. lithosperma | Bauters K. & Coenen J. 2013-026 | USA | GENT | MG708927 |  | MG708669 | MG708782 |
| SC184 | Scleria | lithosperma var. lithosperma | Bauters K. & Coenen J. 2013-027 | USA | GENT | MG708928 |  | MG708670 | MG708783 |
| SC199 | Scleria | angustifolia | Robinson E.A. 4286 | Zambia | MO | MG708929 | MG708543 | MG708671 | MG708784 |
| SC202 | Scleria | catophylla | Carvalho 4674 | Equatorial Guinea | MO | MG708930 | LN886880 | LN886996 | LN887111 |
| SC207 | Scleria | bequaertii | Robinson E.A. 4260 | Zambia | MO | MG708931 | LN886885 | LN887001 | LN887114 |
| SC210 | Scleria | brownii | Michell C.R. & Deichmann B. 3027 | Australia | MO | MG708932 | LN886887 | LN887003 | LN887116 |
| SC214 | Scleria | depauperata | Raynal J. & Jaffré T. 16510 | New Caledonia | MO | MG708933 | LN886888 | LN887004 | LN887117 |
| SC215 | Scleria | dregeana | Bidgood et al. 5393 | Tanzania | MO | MG708934 | MG708544 | MG708672 | MG708785 |
| SC252 | Scleria | pantadenia | Bidgood et al. 5550 | Tanzania | MO | MG708935 | LN886902 | LN887019 | LN887131 |
| SC253 | Scleria | distans var. distans | Bauters K. & Coenen J. 2013-009 | USA | GENT | MG708936 | MG708545 | MG708673 | MG708786 |
| SC255 | Scleria | lithosperma var. lithosperma | Bauters K. & Coenen J. s.n. | USA | GENT | MG708937 |  |  |  |
| SC259 | Scleria | sphacelata | Bruhl J.J. 2467 | Australia | NE | MG708938 | LN886903 | LN887020 | LN887132 |
| SC263 | Scleria | paupercula | Merrett 990 | Zambia | GENT | MG708939 | MG708546 | MG708674 | MG708787 |
| SC264 | Scleria | pooides | Merrett 1027 | Zambia | GENT | MG708940 | MG708547 | MG708675 | MG708788 |
| SC265 | Scleria | schliebenii | Reekmans 6830 | Burundi | GENT | MG708941 | LN996904 | LN887022 | LN887134 |
| SC266 | Scleria | tricristata | Bidgood et al. 6083 | Tanzania | MO | MG708942 | LN886905 | LN887023 | LN887135 |
| SC267 | Scleria | tricristata | Bidgood et al. 5846 | Tanzania | MO | MG708943 | LN886906 | LN887024 | LN887136 |
| SC268 | Scleria | pantadenia | Bidgood et al. 8133 | Tanzania | MO | MG708944 | LN886907 | LN887025 | LN887137 |
| SC269 | Scleria | glabra | Bingham 7603 | Zambia | GENT | MG708945 | MG708548 | MG708676 | MG708789 |
| SC270 | Scleria | hispidula | Ash J.W. 2126 | Ethiopia | MO | MG708946 | MG708549 | MG708677 | MG708790 |
| SC285 | Scleria | bulbifera | Laegaard S. 16215 | Zimbabwe | GENT |  | MG708550 | MG708678 |  |
| SC286 | Scleria | melanotricha | Porembski 2549 | Benin | GENT | MG708947 | MG708551 | MG708679 |  |
| SC287 | Scleria | pergracilis var. brachystachys | Lisowski 442 | Democratic Republic of Congo | GENT | MG708948 | MG708552 | MG708680 | MG708791 |
| SC288 | Scleria | delicatula | Robinson 4555 | Zambia | NY | MG708949 | MG708553 | MG708681 | MG708792 |
| SC290 | Scleria | tenella | Ja-jar 4691 | Guyana | GENT | MG708950 | MG708554 | MG708682 | MG708793 |
| SC291 | Scleria | catophylla | Hess H.&E. 52/505 | Angola | GENT | MG708951 | MG708555 | MG708683 | MG708794 |
| SC292 | Scleria | calcicola | Robinson E.A. 3676 | Zambia | NY | MG708952 | MG708556 | MG708684 | MG708795 |
| SC293 | Scleria | composita | Beck St. G. 13175 | Bolivia | GENT | MG708953 | MG708557 | MG708685 | MG708796 |
| SC294 | Scleria | fulvipilosa | Robinson 6324 | Zambia | NY | MG708954 | MG708558 | MG708686 | MG708797 |
| SC296 | Scleria | leptostachya | Irwin et al. 11690 | Brazil | GENT | MG708955 | MG708559 | MG708687 | MG708798 |
| SC297 | Scleria | leptostachya | Irwin 25930 | Brazil | GENT | MG708956 | MG708560 | MG708688 | MG708799 |
| SC298 | Scleria | hilsenbergii | Dhondt 7 | Madagascar | GENT |  | MG708561 | MG708689 |  |
| SC299 | Scleria | polyrrhiza | Robinson 4921 | Zambia | GENT | MG708957 | MG708562 | MG708690 | MG708800 |
| SC301 | Scleria | veseyfitzgeraldii | Smith 3774 | Botswana | GENT | MG708958 | MG708563 | MG708691 | MG708801 |
| SC303 | Scleria | glabra | Robinson 5115 | Zambia | MO | MG708959 |  | MG708692 | MG708802 |
| SC304 | Scleria | richardsiae | Faden R.B. et al. 96/245 | Tanzania | BR | MG708960 | MG708564 | MG708693 | MG708803 |
| SC305 | Scleria | robinsoniana | Gomar A.S. 343 | Guinea | BR | MG708961 | MG708565 | MG708694 | MG708804 |
| SC306 | Scleria | schliebenii | Senterre et al. 4740 | Democratic Republic of Congo | BR | MG708962 | MG708566 | MG708695 | MG708805 |
| SC307 | Scleria | veseyfitzgeraldii | Robinson 3537 | Zambia | BR | MG708963 | MG708567 | MG708696 | MG708806 |
| SC308 | Scleria | richardsiae | Robinson 3099 | Zambia | BR | MG708964 | MG708568 | MG708697 | MG708807 |
| SC309 | Scleria | composita | Troels Myndel Pedersen 4510 | Argentinia | BR | MG708965 | MG708569 | MG708698 | MG708808 |
| SC310 | Scleria | melanotricha | Porembski 575 | Ivory Coast | GENT | MG708966 |  |  |  |
| SC311 | Scleria | spec. nov. ('ankaratrensis') | Larridon et al. 2010-0340 | Madagascar | GENT | MG708967 | MG708570 | MG708699 | MG708809 |
| SC313 | Scleria | bourgeaui | Pringle 1686 | Mexico | BR |  | MG708571 |  |  |
| SC315 | Scleria | perpusilla | Chermezon 18433 | Madagascar | BR | MG708968 |  |  |  |
| SC317 | Scleria | woodii | Goetghebeur 5030 | Cameroon | GENT | MG708969 | MG708572 | MG708700 | MG708810 |
| SC318 | Scleria | interrupta | Jansen-Jacobs M.J. s.n. ? | Guyana | GENT | MG708970 | MG708573 | MG708701 | MG708811 |
| SC319 | Scleria | verticillata | Umbach 198 | USA | GENT | MG708971 | MG708574 |  | MG708812 |
| SC321 | Scleria | longispiculata | Hess H.&E. 52/580 | Angola | GENT | MG708972 | MG708575 | MG708702 | MG708813 |
| SC323 | Scleria | lithosperma var. lithosperma | Bauters K. & Coenen J. 2013-024 | USA | GENT | MG708973 |  |  |  |
| SC324 | Scleria | polyrrhiza | E. A. Robinson 2124 | Zambia | NY | MG708974 | MG708576 | MG708703 | MG708814 |
| SC326 | Scleria | woodii | S. Bidgood et al. 5202 | Tanzania | GENT | MG708975 | MG708577 | MG708704 | MG708815 |
| SC328 | Scleria | paupercula | Robinson 5767 | Zambia | NY | MG708976 | MG708578 | MG708705 | MG708816 |
| SC329 | Scleria | paupercula | Robinson 5762 | Zambia | NY | MG708977 | MG708579 |  |  |
| SC330 | Scleria | paupercula | Robinson 4063 | Zambia | NY | MG708978 |  |  |  |
| SC331 | Scleria | richardsiae | R. B. Faden et al. 96/245 | Tanzania | BR |  | MG708580 |  | MG708817 |
| SC333 | Scleria | erythrorrhiza | S. Pawek 10890 | Malawi | MO | MG708979 | MG708581 | MG708706 | MG708818 |
| SC334 | Scleria | richardsiae | H.M. Richards 14254 | Tanzania | NY |  |  | MG708707 | MG708819 |
| SC335 | Scleria | zambesica | E.A. Robinson 6578 | Zambia | NY | MG708980 | MG708582 | MG708708 | MG708820 |
| SC345 | Scleria | dregeana | Bauters K. 2015-007 | Zambia | GENT | MG708981 | MG708583 | MG708709 | MG708821 |
| SC349 | Scleria | pergracilis var. brachystachys | Bauters K. 2015-052 | Zambia | GENT | MG708982 | MG708584 | MG708710 | MG708822 |
| SC350 | Scleria | suaveolens | Bauters K. 2015-053 | Zambia | GENT | MG708983 | MG708585 | MG708711 | MG708823 |
| SC351 | Scleria | flexuosa | Bauters K. 2015-057 | Zambia | GENT | MG708984 | MG708586 | MG708712 | MG708824 |
| SC355 | Scleria | schliebenii | Bauters K. 2015-071 | Zambia | GENT | MG708985 | MG708587 | MG708713 | MG708825 |
| SC358 | Scleria | rehmannii | Bauters K. 2015-107 | Zambia | GENT | MG708986 | MG708588 | MG708714 | MG708826 |
| SC359 | Scleria | rehmannii | Bauters K. 2015-109 | Zambia | GENT |  | MG708589 | MG708715 | MG708827 |
| SC361 | Scleria | rehmannii | Bauters K. 2015-113 | Zambia | GENT | MG708987 | MG708590 | MG708716 |  |
| SC362 | Scleria | dregeana | Bauters K. 2015-116 | Zambia | GENT |  | MG708591 | MG708717 |  |
| SC363 | Scleria | rehmannii | Bauters K. 2015-118 | Zambia | GENT | MG708988 | MG708592 | MG708718 |  |
| SC364 | Scleria | pooides | Bauters K. 2015-119 | Zambia | GENT | MG708989 | MG708593 | MG708719 | MG708828 |
| SC365 | Scleria | pooides | Bauters K. 2015-122 | Zambia | GENT | MG708990 | MG708594 | MG708720 |  |
| SC367 | Scleria | pooides | Bauters K. 2015-129 | Zambia | GENT | MG708991 | MG708595 | MG708721 |  |
| SC368 | Scleria | pergracilis var. brachystachys | Bauters K. 2015-139 | Zambia | GENT | MG708992 | MG708596 | MG708722 | MG708829 |
| SC369 | Scleria | rehmannii | Bauters K. 2015-140 | Zambia | GENT | MG708993 | MG708597 | MG708723 |  |
| SC371 | Scleria | suaveolens | Bauters K. 2015-150 | Zambia | GENT | MG708994 | MG708598 | MG708724 | MG708830 |
| SC372 | Scleria | pergracilis var. brachystachys | Bauters K. 2015-151 | Zambia | GENT | MG708995 | MG708599 | MG708725 | MG708831 |
| SC373 | Scleria | flexuosa | Bauters K. 2015-152 | Zambia | GENT | MG708996 | MG708600 | MG708726 | MG708832 |
| SC376 | Scleria | dregeana | Bauters K. 2015-159 | Zambia | GENT | MG708997 | MG708601 | MG708727 | MG708833 |
| SC378 | Scleria | bulbifera | Bauters K. 2015-163 | Zambia | GENT |  | MG708602 | MG708728 | MG708834 |
| SC379 | Scleria | paupercula | Bauters K. 2015-167 | Zambia | GENT | MG708998 |  | MG708729 | MG708835 |
| SC381 | Scleria | bequaertii | Bauters K. 2015-171 | Zambia | GENT | MG708999 | MG708603 | MG708730 | MG708836 |
| SC382 | Scleria | paupercula | Bauters K. 2015-172 | Zambia | GENT | MG709000 | MG708604 | MG708731 | MG708837 |
| SC383 | Scleria | pergracilis var. brachystachys | Bauters K. 2015-178 | Zambia | GENT | MG709001 | MG708605 |  | MG708838 |
| SC384 | Scleria | dregeana | Bauters K. 2015-184 | Zambia | GENT | MG709002 | MG708606 | MG708732 | MG708839 |
| SC386 | Scleria | pooides | Bauters K. 2015-189 | Zambia | GENT | MG709003 | MG708607 | MG708733 | MG708840 |
| SC387 | Scleria | paupercula | Bauters K. 2015-195 | Zambia | GENT | MG709004 | MG708608 | MG708734 | MG708841 |
| SC389 | Scleria | welwitschii | Bauters K. 2015-198 | Zambia | GENT | MG709005 | MG708609 |  | MG708842 |
| SC390 | Scleria | dregeana | Bauters K. 2015-200 | Zambia | GENT | MG709006 | MG708610 |  | MG708843 |
| SC391 | Scleria | pooides | Bauters K. 2015-207 | Zambia | GENT |  | MG708611 |  |  |
| SC433 | Scleria | schliebenii | Richards M. 18887 | Zambia | GENT | MG709007 | MG708612 | MG708735 | MG708844 |
| SC434 | Scleria | veseyfitzgeraldii | Robinson 5513 | Zambia | GENT | MG709008 | MG708613 | MG708736 | MG708845 |
| SC435 | Scleria | spec. nov. ('pedicellata') | Ngok Banak 1736 | Togo | MO | MG709009 | MG708614 | MG708737 | MG708846 |
| SC436 | Scleria | spec. nov. ('liberica') | Adam 29622 | Liberia | MO | MG709010 | MG708615 | MG708738 | MG708847 |
| SC437 | Scleria | angustifolia | Robinson 6623 | Zambia | GENT | MG709011 | MG708616 | MG708739 | MG708848 |
| SC438 | Scleria | bulbifera | Richards M. 16893 | Zambia | GENT | MG709012 |  | MG708740 | MG708849 |
| SC439 | Scleria | hispidula | Robinson 6535 | Zambia | GENT | MG709013 | MG708617 | MG708741 | MG708850 |
| SC440 | Scleria | hispidula | Robinson 6454 | Zambia | GENT | MG709014 | MG708618 | MG708742 | MG708851 |
| SC441 | Scleria | bequaertii | Robinson 5537 | Zambia | GENT | MG709015 | MG708619 | MG708743 | MG708852 |
| SC442 | Scleria | welwitschii | Robinson 5714 | Zambia | GENT | MG709016 | MG708620 | MG708744 | MG708853 |
| SC443 | Scleria | angustifolia | Robinson 4286 | Zambia | NY | MG709017 | MG708621 | MG708745 | MG708854 |
| SC444 | Scleria | calcicola | Robinson 4630 | Zambia | NY | MG709018 | MG708622 | MG708746 | MG708855 |
| SC445 | Scleria | hirtella | Piepenbring 2625 | Guyana | GENT | MG709019 | MG708623 | MG708747 | MG708856 |
| SC446 | Scleria | laxiflora | Bidgood et al. 842 | Tanzania | MO | MG709020 | MG708624 | MG708748 | MG708857 |
| SC447 | Scleria | pusilla | Jansen-Jacobs 2637 | Guyana | GENT | MG709021 | MG708625 | MG708749 | MG708858 |
| SC448 | Scleria | pulchella | Hess H.&E. 52/1745 | Angola | GENT | MG709022 | MG708626 | MG708750 | MG708859 |
| SC449 | Scleria | melanotricha | Porembski 362 | Ivory Coast | GENT |  | MG708627 | MG708751 | MG708860 |
| SC450 | Scleria | tricholepis | Drummond 7438 | Zambia | L |  | MG708628 |  | MG708861 |
| SC451 | Scleria | spec. nov. ('maypurensis') | Gröger A. 1103 | Venezuela | GENT |  | MG708629 |  | MG708862 |
| SC452 | Scleria | flexuosa | Goetghebeur 9137 | Zimbabwe | GENT | MG709023 | MG708630 | MG708752 |  |
| SC453 | Scleria | burchellii | Irwin 24394 | Brazil | MO |  | MG708631 |  | MG708863 |
| SC454 | Scleria | spec. nov. ('pseudohispidior') | Friis 7889 | Ethiopia | L | MG709024 | MG708632 | MG708753 | MG708864 |
| SC455 | Scleria | hispidior | Gilbert 927 | Ethiopia | L |  | MG708633 |  | MG708865 |
| SC457 | Scleria | cuyabensis | Zardini 48117 | Paraguay | GENT |  |  |  | MG708866 |
