# Supplementary figures and images for: Molecular phylogenetic study of Scleria subgenus Hypoporum (Sclerieae, Cyperoideae, Cyperaceae) reveals several species new to science
Source: PLoS One. 2018 Sep 27;13(9):e0203478. doi: 10.1371/journal.pone.0203478 (PMC6160245; doi:10.1371/journal.pone.0203478)

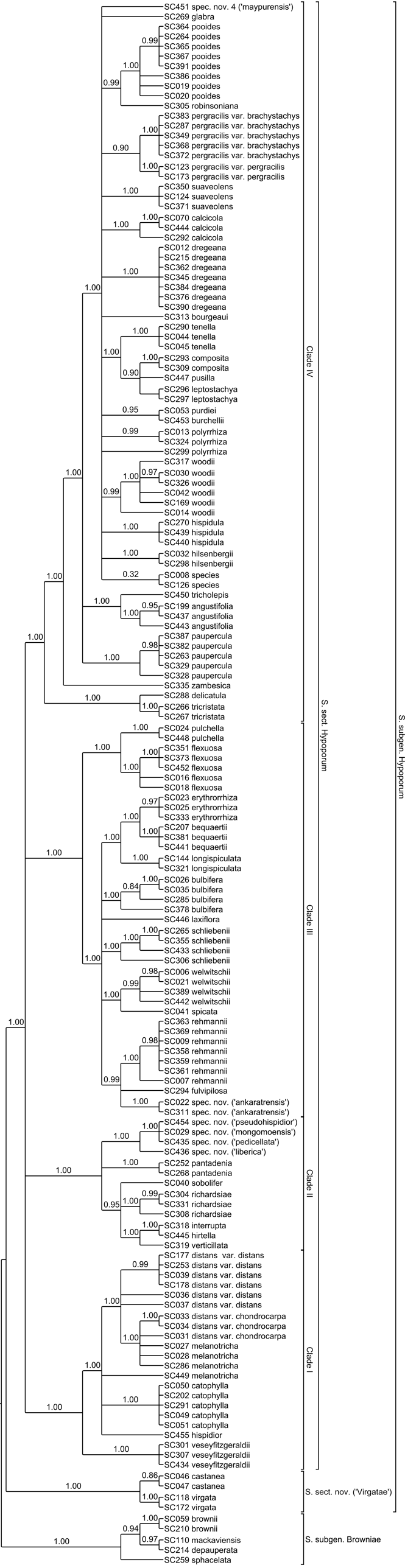

Supplement: S1 Fig — Posterior probabilities indicated on the respective branches when equal or higher than 0.90. (PDF) [file pone.0203478.s002.pdf]

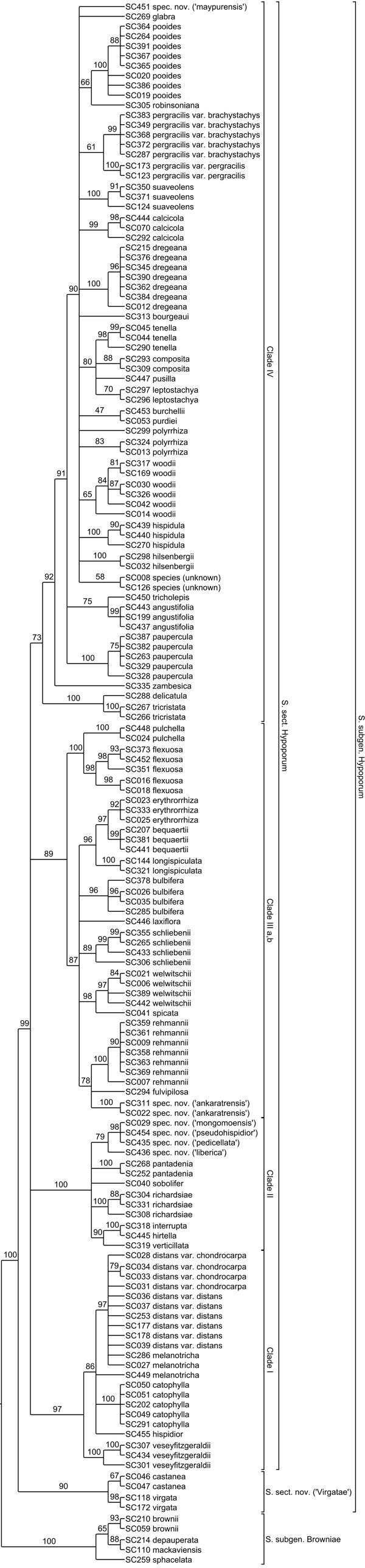

Supplement: S2 Fig — (PDF) [file pone.0203478.s003.pdf]

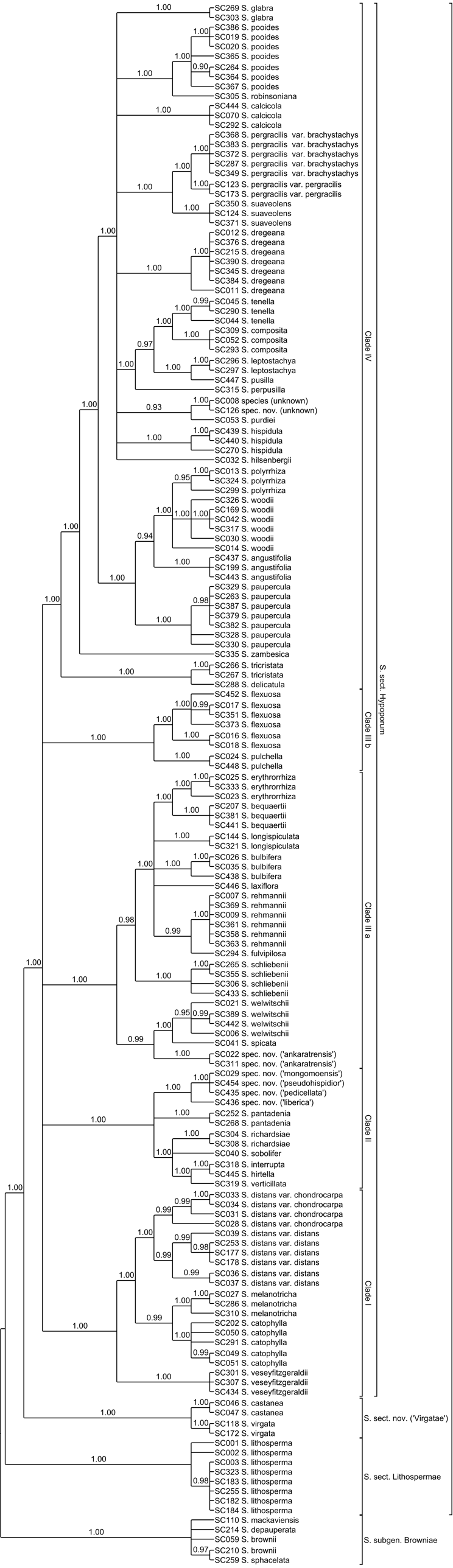

Supplement: S3 Fig — Posterior probabilities indicated on the respective branches when equal or higher than 0.90. (PDF) [file pone.0203478.s004.pdf]

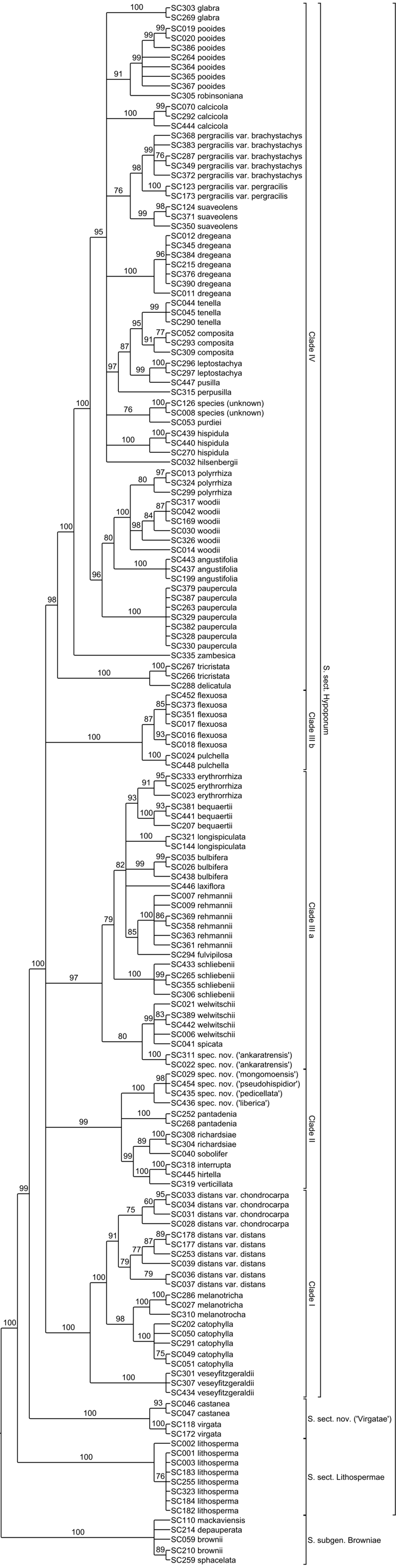

Supplement: S4 Fig — (PDF) [file pone.0203478.s005.pdf]

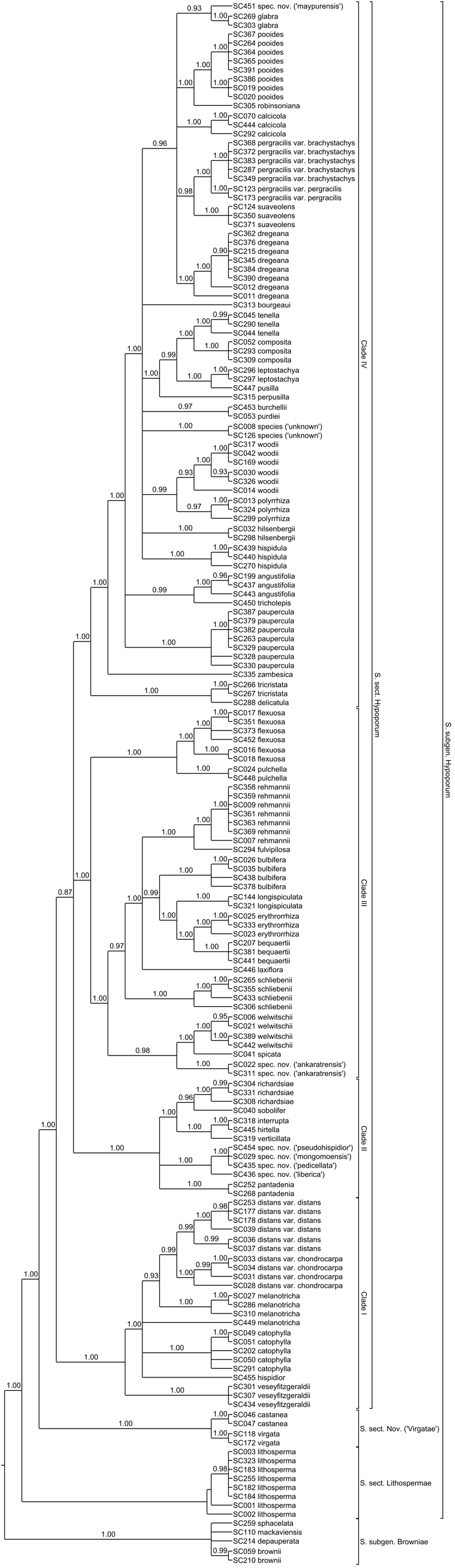

Supplement: S5 Fig — Posterior probabilities indicated on the respective branches when equal or higher than 0.90. (PDF) [file pone.0203478.s006.pdf]

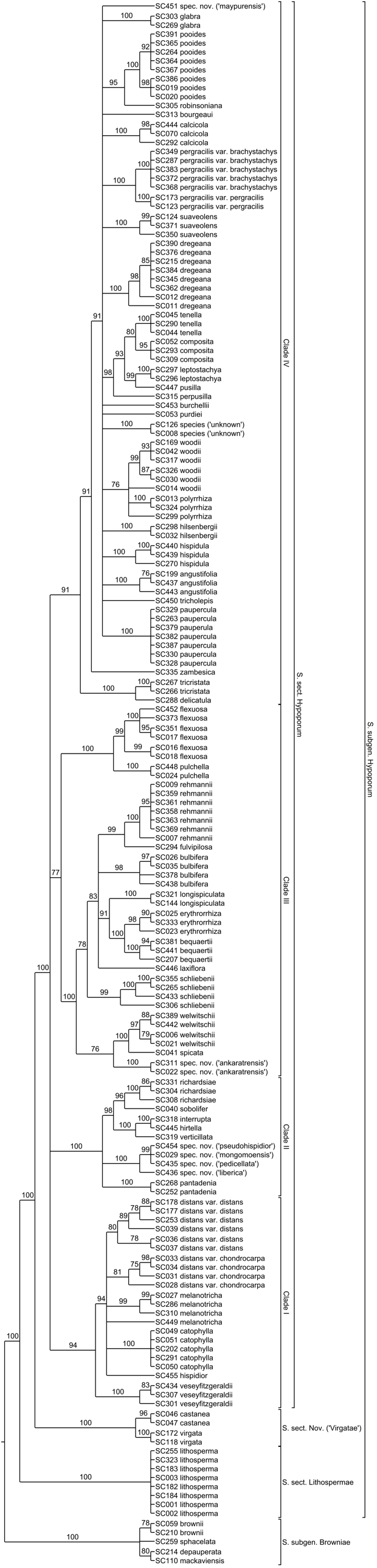

Supplement: S6 Fig — (PDF) [file pone.0203478.s007.pdf]

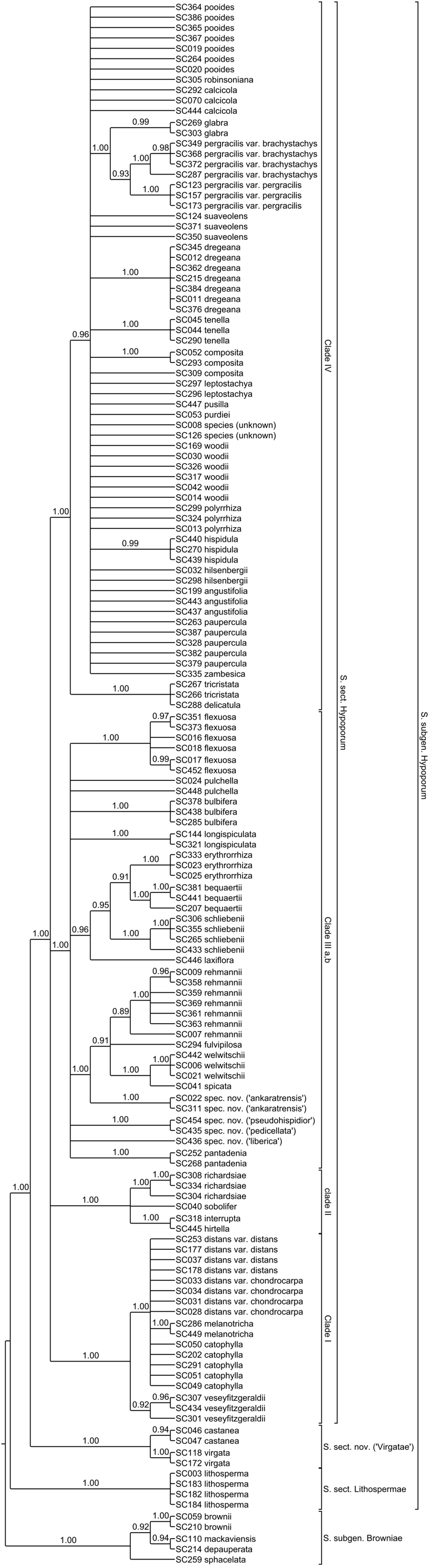

Supplement: S7 Fig — Posterior probabilities indicated on the respective branches when equal or higher than 0.90. (PDF) [file pone.0203478.s008.pdf]

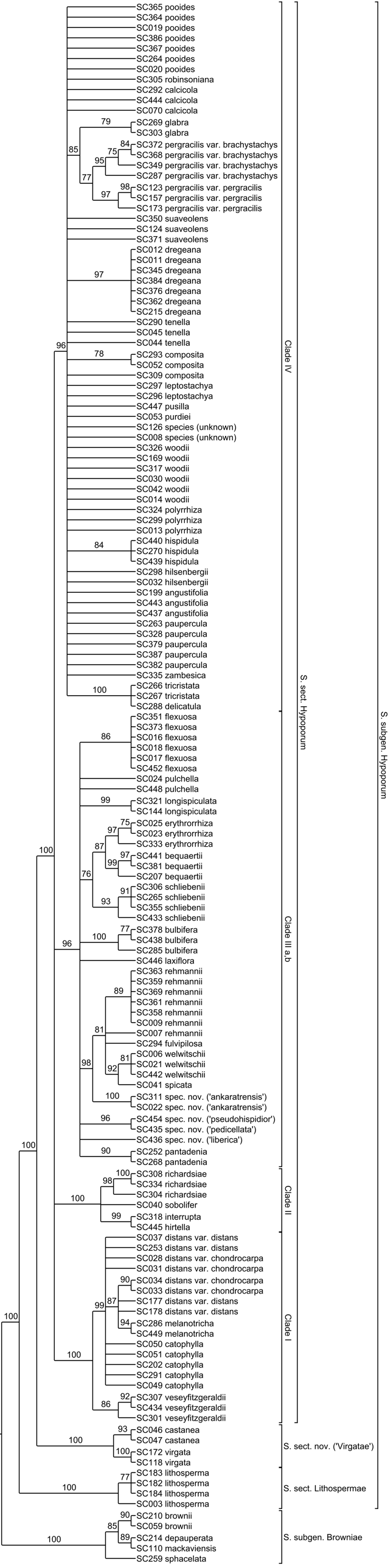

Supplement: S8 Fig — (PDF) [file pone.0203478.s009.pdf]

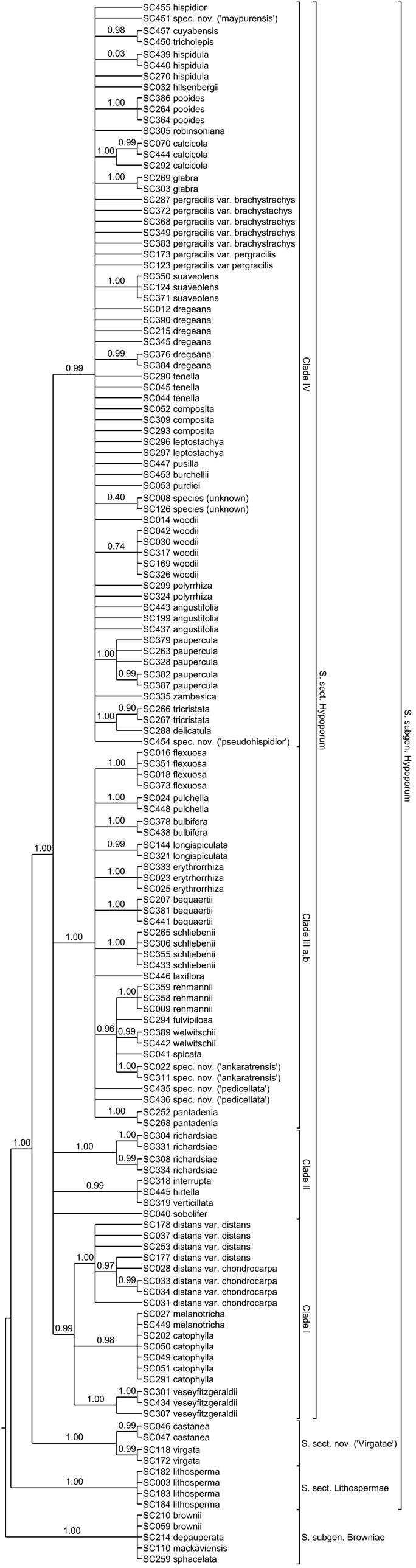

Supplement: S9 Fig — Posterior probabilities indicated on the respective branches when equal or higher than 0.90. (PDF) [file pone.0203478.s010.pdf]

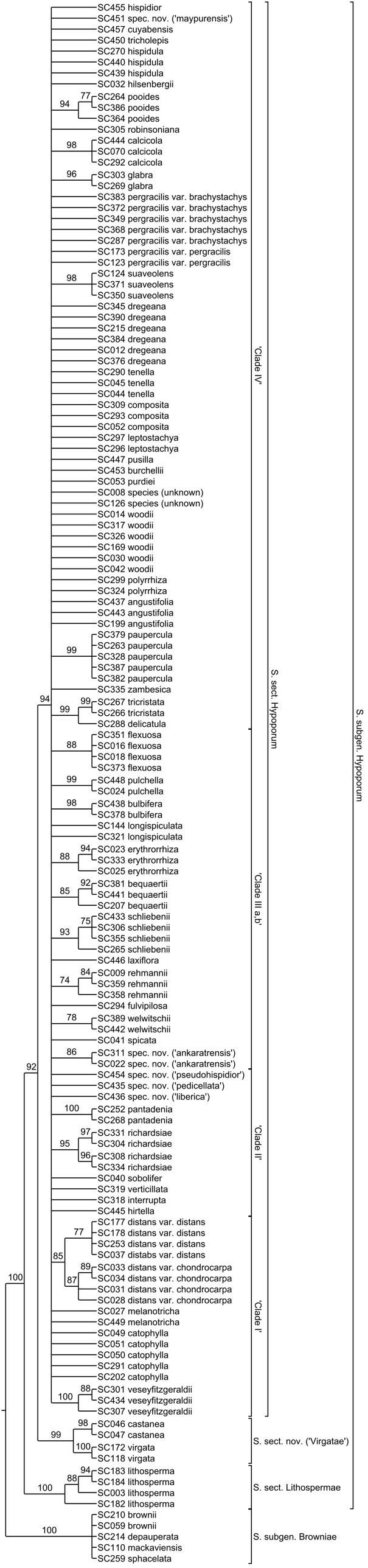

Supplement: S10 Fig — (PDF) [file pone.0203478.s011.pdf]

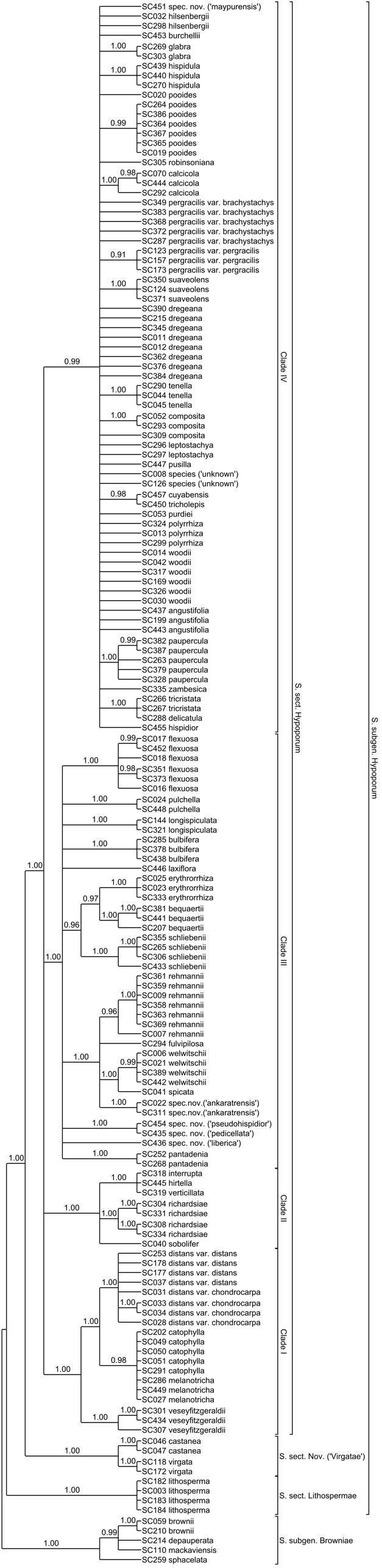

Supplement: S11 Fig — Posterior probabilities indicated on the respective branches when equal or higher than 0.90. (PDF) [file pone.0203478.s012.pdf]

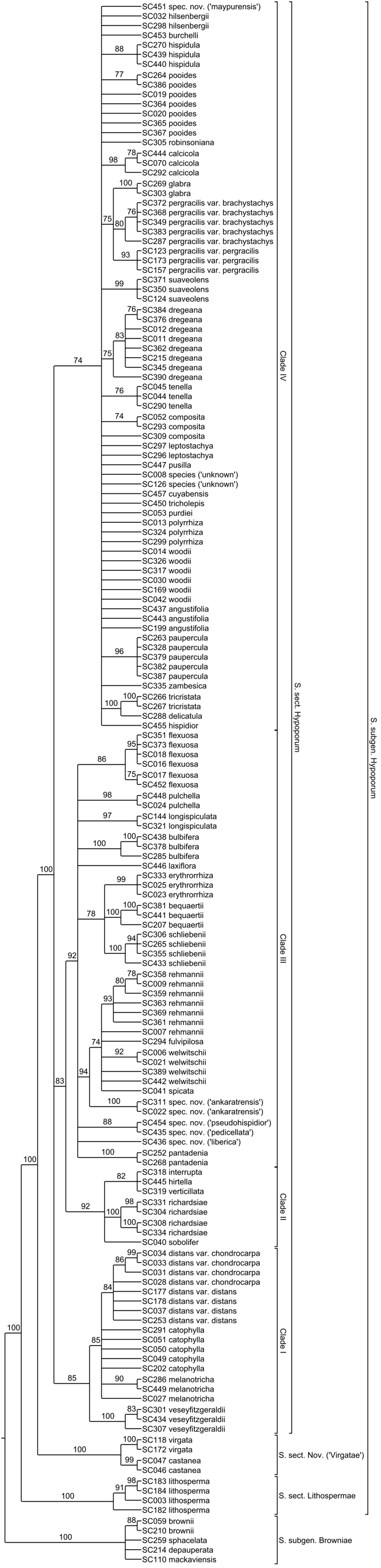

Supplement: S12 Fig — (PDF) [file pone.0203478.s013.pdf]
